# Supplementary material for: A druggable copper-signalling pathway that drives inflammation
Source: Nature. 2023 Apr 26;617(7960):386–94. doi: 10.1038/s41586-023-06017-4 (PMC10131557; doi:10.1038/s41586-023-06017-4)

**Supplementary Information**

**This document includes:**

Supplementary table legends

Sequences for RNA interference and genome editing experiments

Flow cytometry gating strategies

Full western blots

NMR spectra

**Supplementary Table 1 | Quantitative metabolomics analysis of mitochondrial cell extracts**

**Supplementary Table 2 | Quantitative metabolomics analysis of total cell extracts**

**Supplementary Table 3 | RNA-seq analysis of MDM**

Differential gene expression was assessed with the limma/voom framework. *P* values were corrected for multiple testing with the Benjamini-Hochberg procedure.

**Supplementary Table 4 | RNA-seq analysis of inflammatory macrophages**

Differential gene expression was assessed with the limma/voom framework. *P* values were corrected for multiple testing with the Benjamini-Hochberg procedure.

**Supplementary Table 5 | GO terms of RNA analysis of genes upregulated in inflammatory macrophages**

GO enrichment was assessed with the enrichGO method from clusterProfiler. *P* values were corrected for multiple testing with the Benjamini-Hochberg procedure.

**Supplementary Table 6 | Quantitative mass-spectrometry-based proteomics in MDM**

**Supplementary Table 7 | ChIP-seq analysis of MDM**

**Supplementary Table 8 | GO terms of RNA analysis of genes downregulated in MDM treated with LCC-12 during activation**

GO enrichment was assessed with the enrichGO method from clusterProfiler. *P* values were corrected for multiple testing with the Benjamini-Hochberg procedure.

**Supplementary Table 9 | RNA-seq analysis of aMDM under CD44 knock out conditions**

**Supplementary Table 10 | RNA-seq analysis of SPM**

**Supplementary Table 11 | RNA-seq analysis of lung tissues from SARS-CoV-2-infected mice**

Differential gene expression was assessed with the limma/voom framework. *P* values were corrected for multiple testing with the Benjamini-Hochberg procedure.

**Supplementary Table 12 | GO terms of RNA analysis of genes downregulated in lung tissues from SARS-CoV-2-infected mice treated with LCC-12**

GO enrichment was assessed with the enrichGO method from clusterProfiler. *P* values were corrected for multiple testing with the Benjamini-Hochberg procedure.

**SMARTpools used for RNA interference:**

| Target gene: *CD44* (protein: CD44) | |
| --- | --- |
| L-009999-00-0020, ON-TARGETplus Human CD44 (960) siRNA (Dharmacon) | |
| Sequence 1: | GAAUAUAACCUGCCGCUUU |
| Sequence 2: | CAAGUGGACUCAACGGAGA |
| Sequence 3: | CGAAGAAGGUGUGGGCAGA |
| Sequence 4: | GAUCAACAGUGGCAAUGGA |
| Target gene: *SLC31A1* (protein: CTR1) | |
| L-007531-02-0020, ON-TARGETplus Human SLC31A1 (1317) siRNA (Dharmacon) | |
| Sequence 1: | CGAGAGAGCCUGCUGCGUA |
| Sequence 2: | CUCUAGAUGUGAUACGUUA |
| Sequence 3: | UCGCAUGUCUAGUAUAUUA |
| Sequence 4: | UGGCGUGGCCUUAUCGAUU |
| Target gene: *SLC31A2* (protein: CTR2) | |
| L-007532-01-0020, ON-TARGETplus Human SLC31A2 (1318) siRNA (Dharmacon) | |
| Sequence 1: | UUGUAAGCUUUGGGAAUUA |
| Sequence 2: | UUACUGAGCCCAUGACAAU |
| Sequence 3: | GAGCAUCGCCCAUUGGACU |
| Sequence 4: | GUAUGAAGGCAUCAAGGUU |
| Target gene: *SLC11A2* (protein: DMT1) | |
| L-007381-00-0020, ON-TARGETplus Human SLC11A2 (4891) siRNA (Dharmacon) | |
| Sequence 1: | GGACUAGGCUGGCGGAUUG |
| Sequence 2: | AGGAGUACUUCGCCACUUA |
| Sequence 3: | GAUUUAAGUUGCUCUGGAU |
| Sequence 4: | GCUCAGCCAUUGCUAUCAA |
| Target gene: *SLC25A3* (protein: SLC25A3) | |
| L-007484-00-0020, ON-TARGETplus Human SLC25A3 (5250) siRNA (Dharmacon) | |
| Sequence 1: | GGGCAUAUUUAACGGAUUC |
| Sequence 2: | UGGCGCACAUCACUAUAUU |
| Sequence 3: | GCAAUUGUUUCUCACCCUG |
| Sequence 4: | GCCAACACUUUGAGGGAUG |
| Target gene: *SLC25A37* (protein: SLC25A37/Mitoferrin-1) | |
| L-007369-01-0020, ON-TARGETplus Human SLC25A37 (51312) siRNA (Dharmacon) | |
| Sequence 1: | CCACCUAGCCAACGGGAUA |
| Sequence 2: | CAUGACAGCAGGAGCGAUG |
| Sequence 3: | GUACACAAGUAUCUACGGA |
| Sequence 4: | GGUCGGAGGACUACGAGAA |
| Target gene: *TFRC* (protein: TFR1) | |
| Sequence 1: | GAAUGGAUCUAUAGUGAUU |
| Sequence 2: | GAUAAGAACGGUAGACUUG |
| Sequence 3: | GUAAACUGGUCCAUGCUAA |
| Sequence 4: | CUGAAUGGCUAGAGGGAUA |

**Sequences used for genome editing:**

| CD44 sgRNA ((Edit-R Human Synthetic CD44, set of 3, target sequences) |
| --- |
| CGTGGAATACACCTGCAAAG |
| CAATAGCACCTTGCCCACAA |
| TTGCAGTCAACAGTCGAAGA |


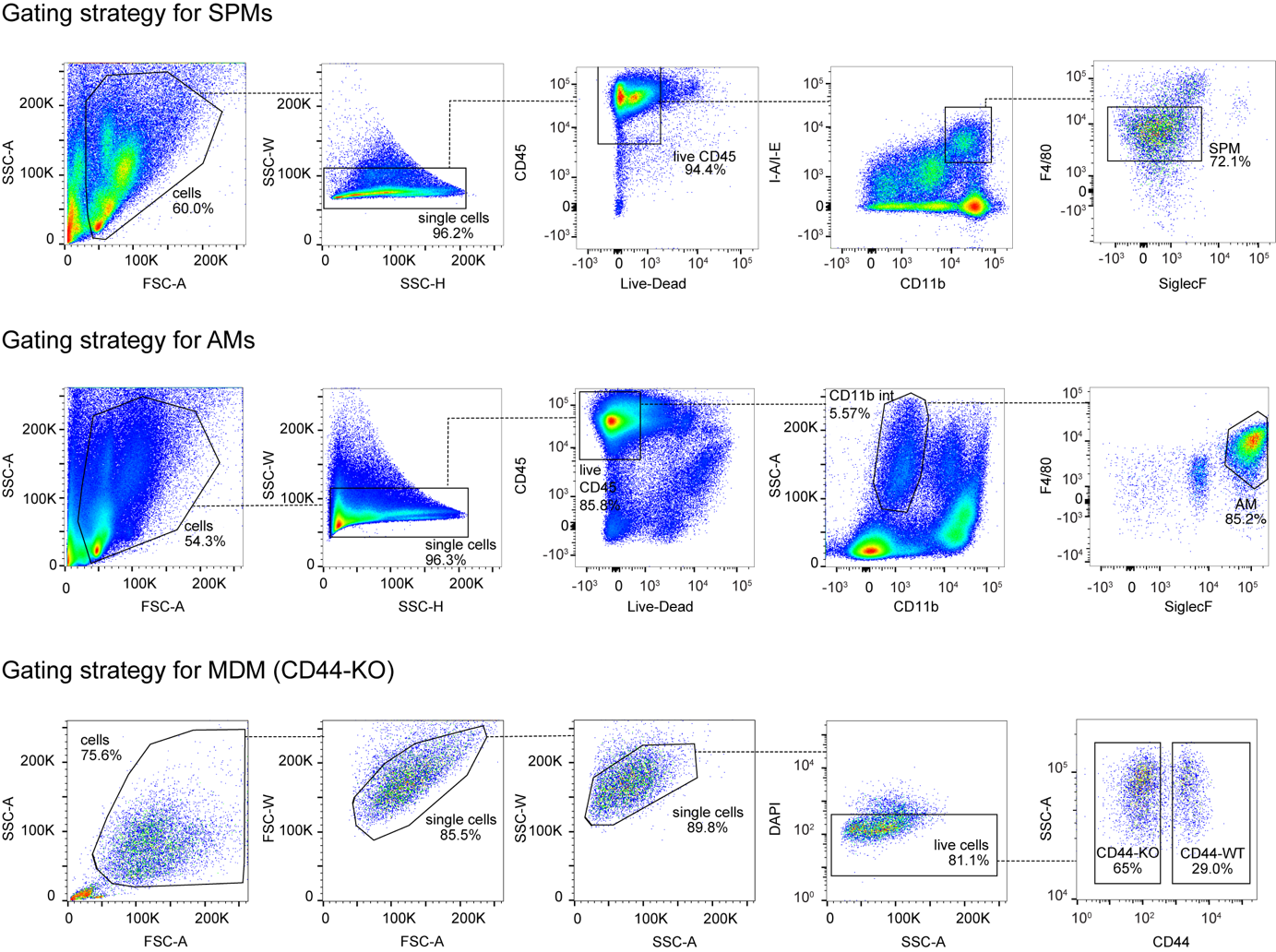


Protein size markers from a protein ladder (PageRuler or PageRuler plus, Thermo Fisher Scientific, 26616 and 26620) are indicated in kilodalton (kDa). Boxes with dotted lines indicate the cropping used for generating the illustrations in the figures.


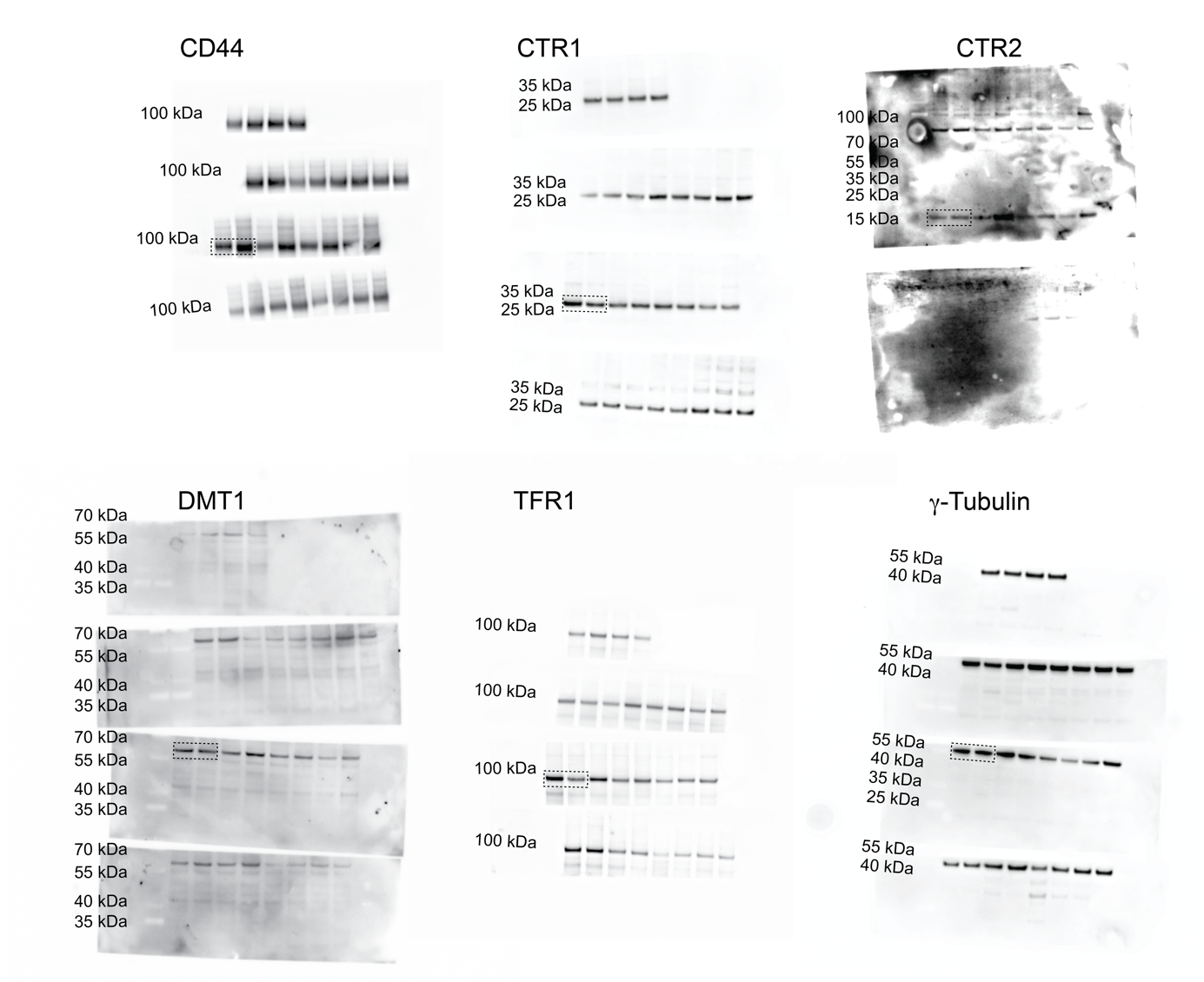


**Figure 1e**

**
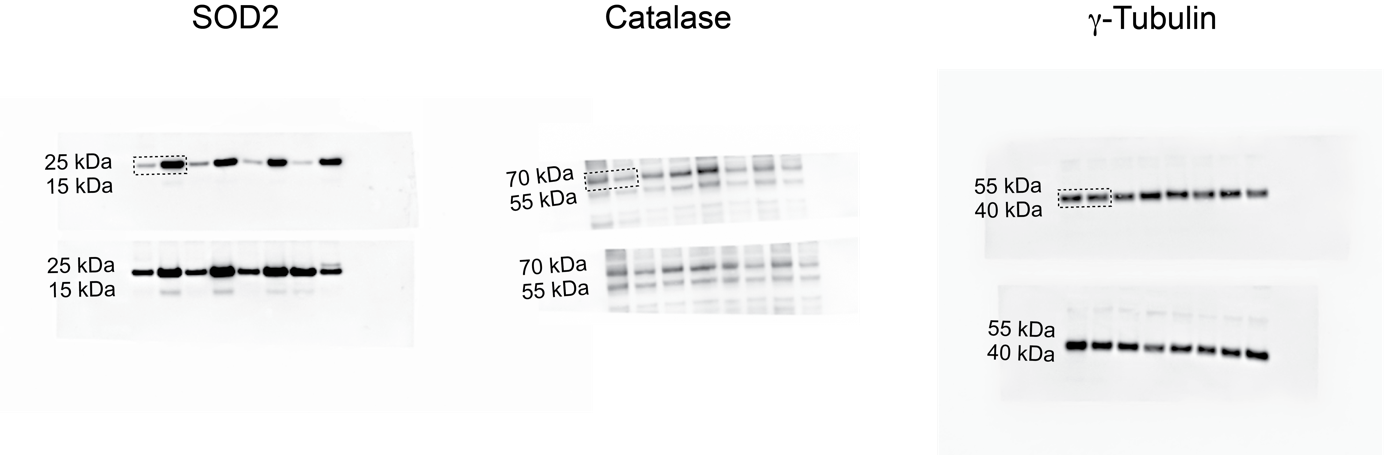
**

**Figure 3b**

| 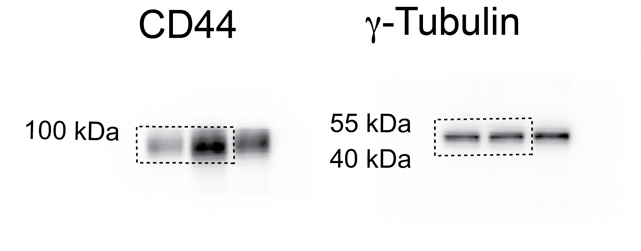 | **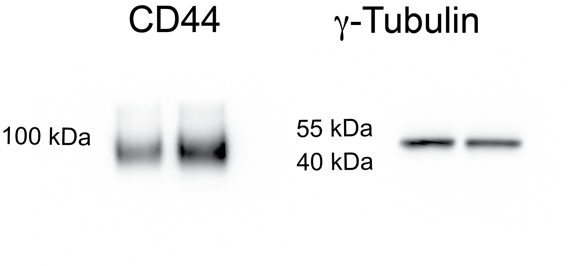** |
| --- | --- |
| **Figure 5b – LPS model** | **Figure 5b – CLP model** |

| 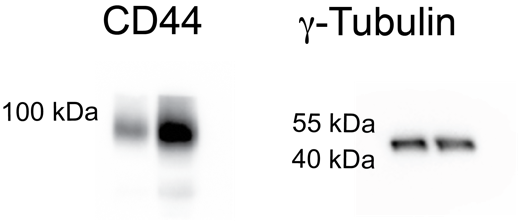 |
| --- |
| **Figure 5b – SARS-CoV-2 model** |


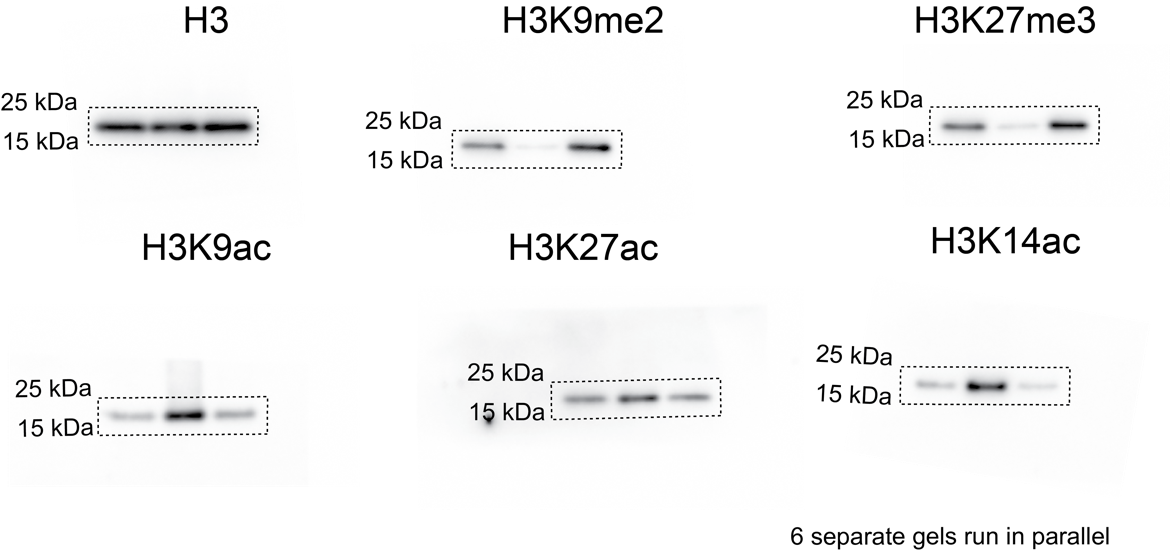


**Figure 5d**

**
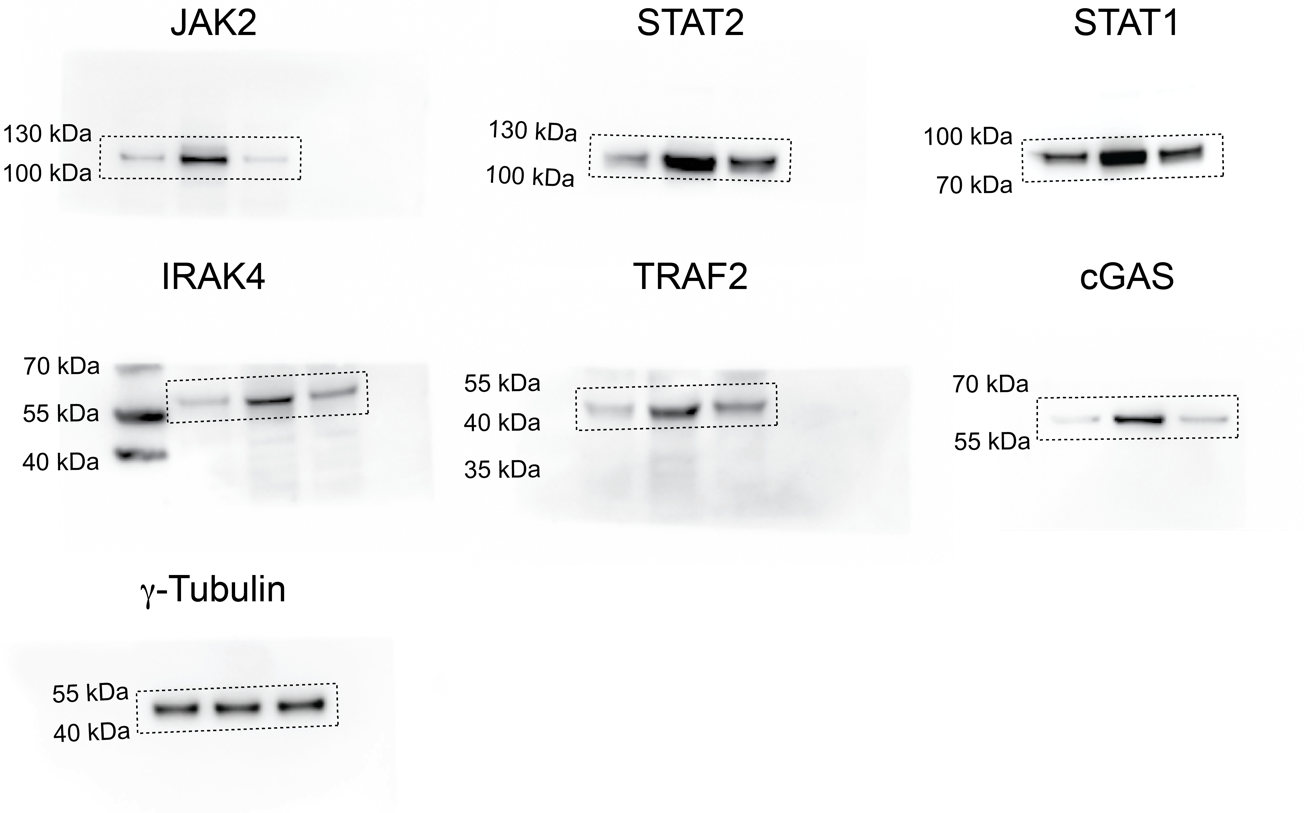
**

**Figure 5f**

**
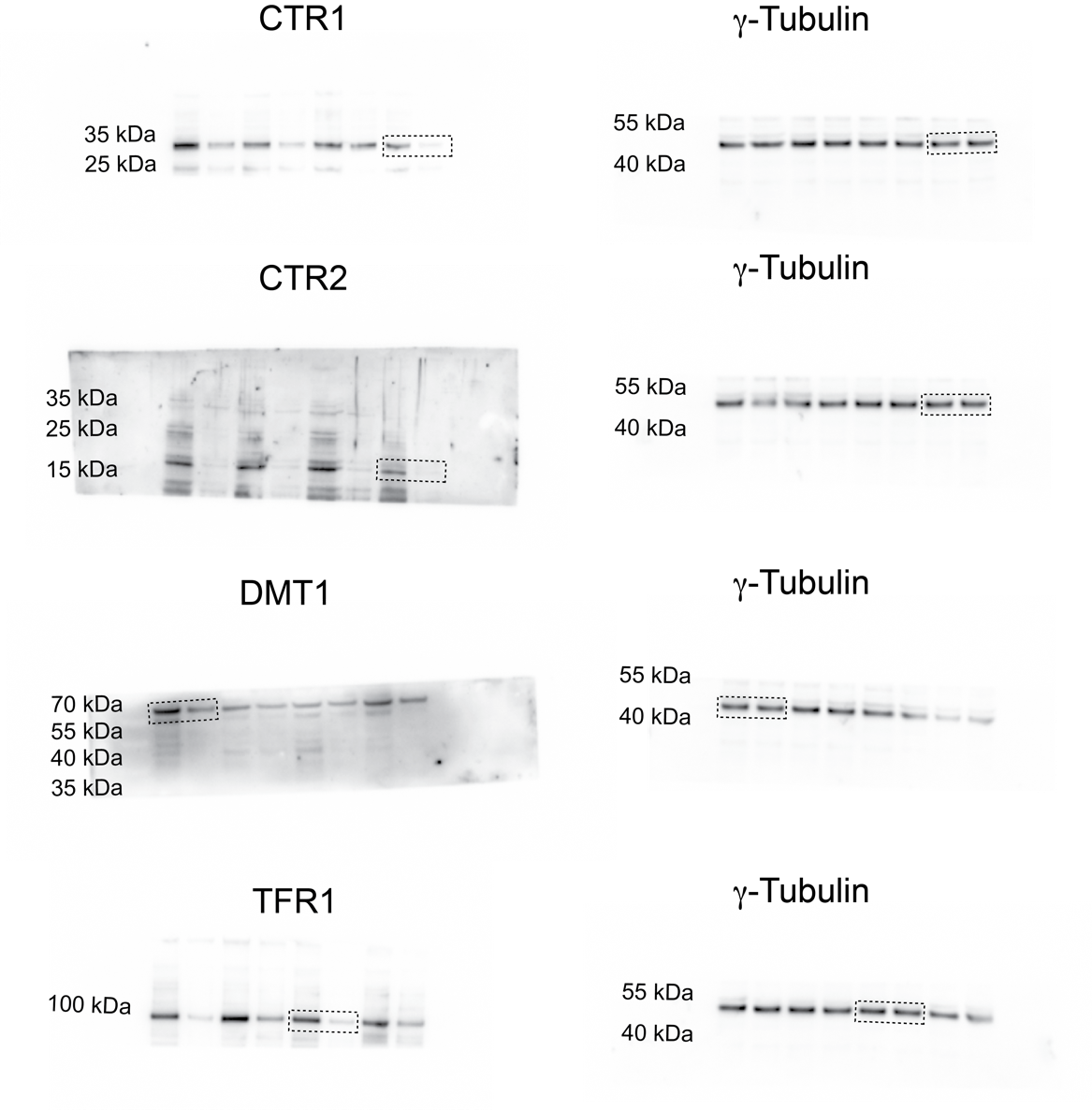
**

**Extended Data Figure 1f**


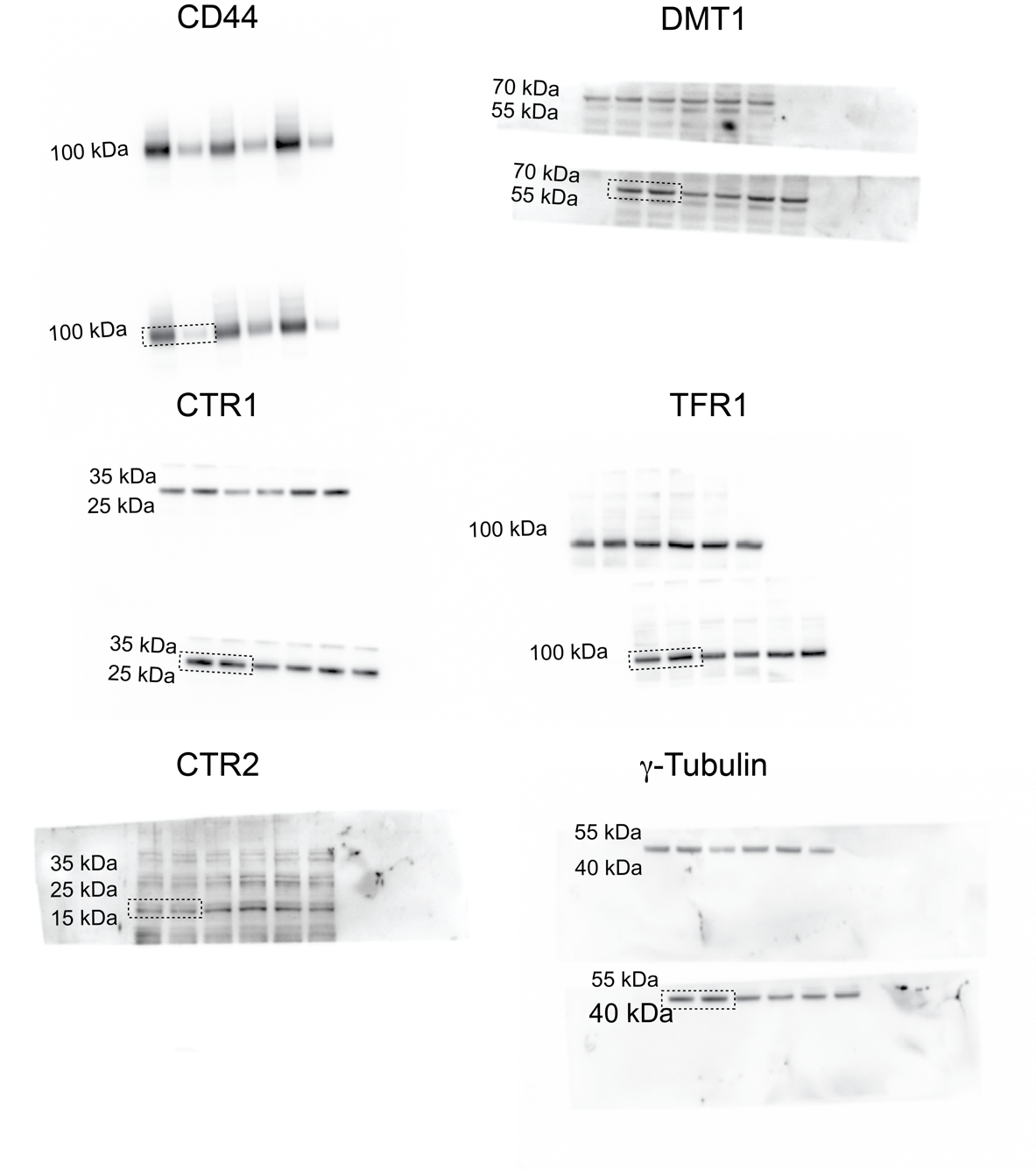


**Extended Data Figure 1g**

­
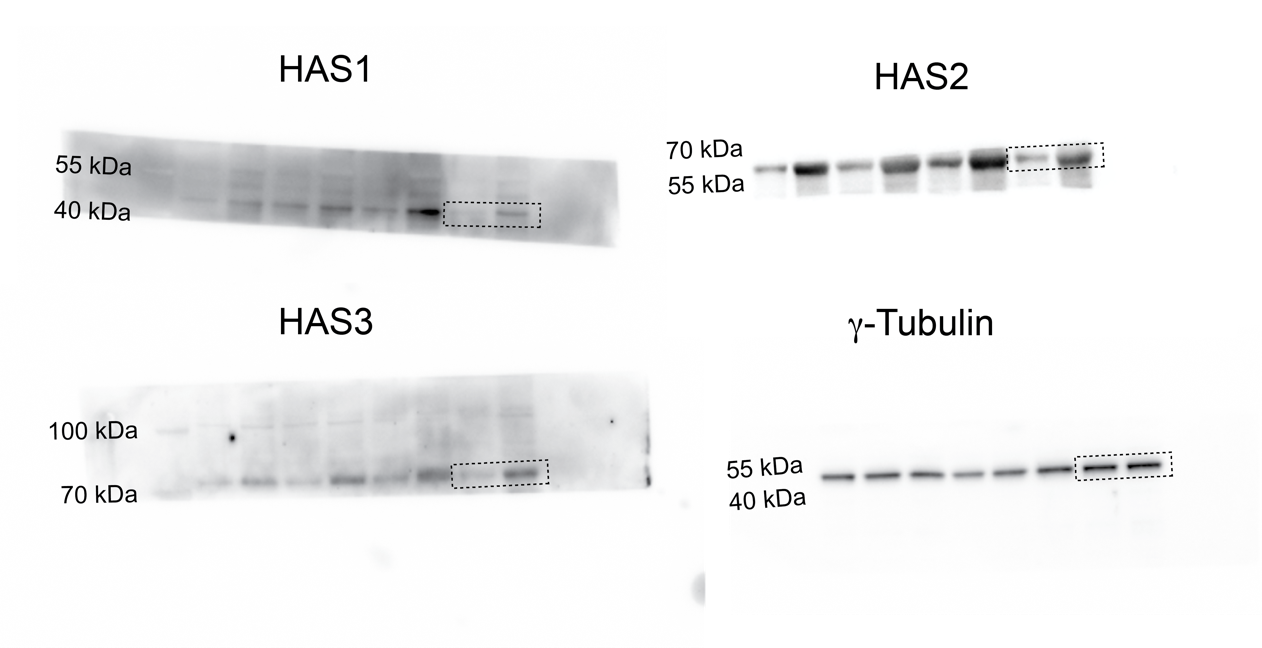


**
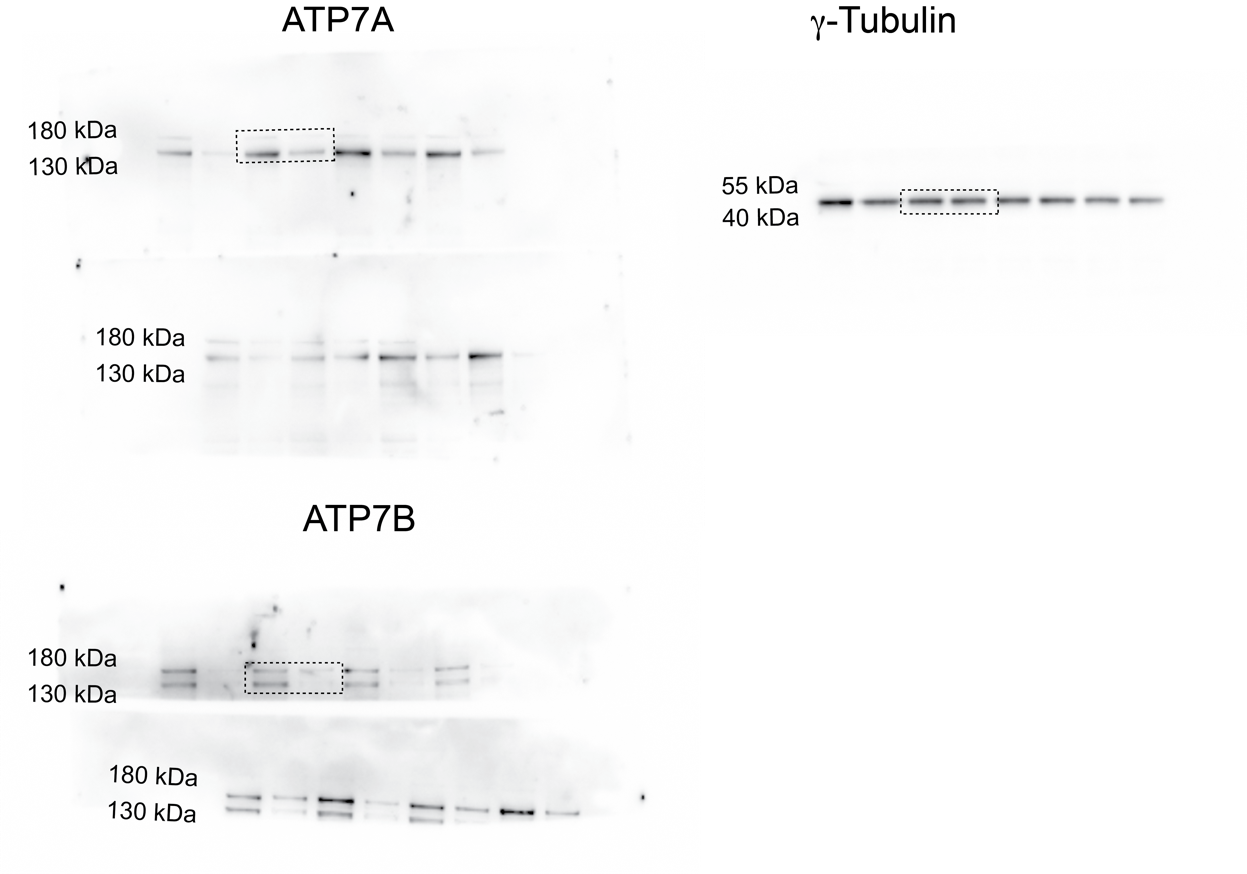
**

**Extended Data Figure 2c**

**
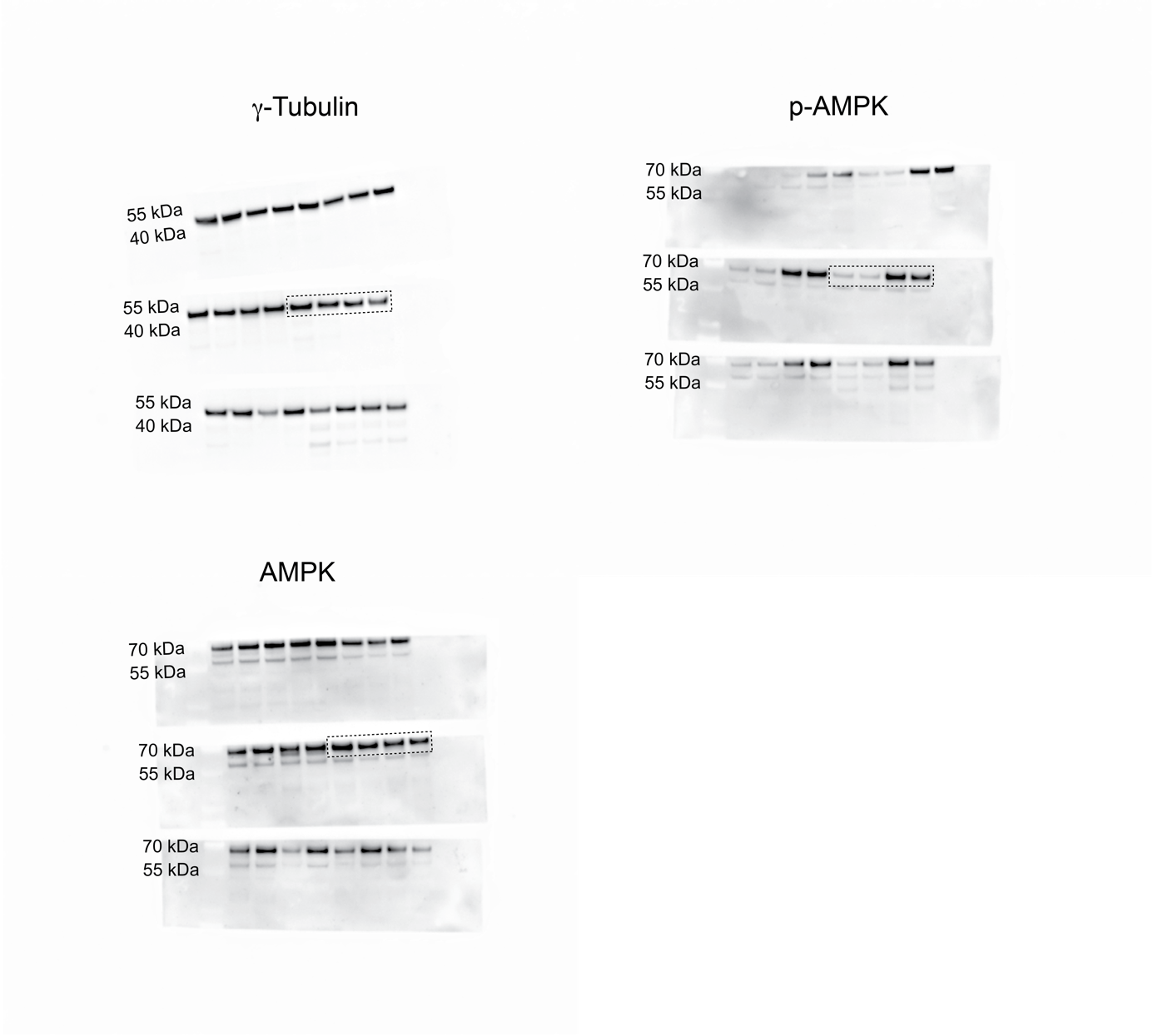
**

**Extended Data Figure 3g**

**
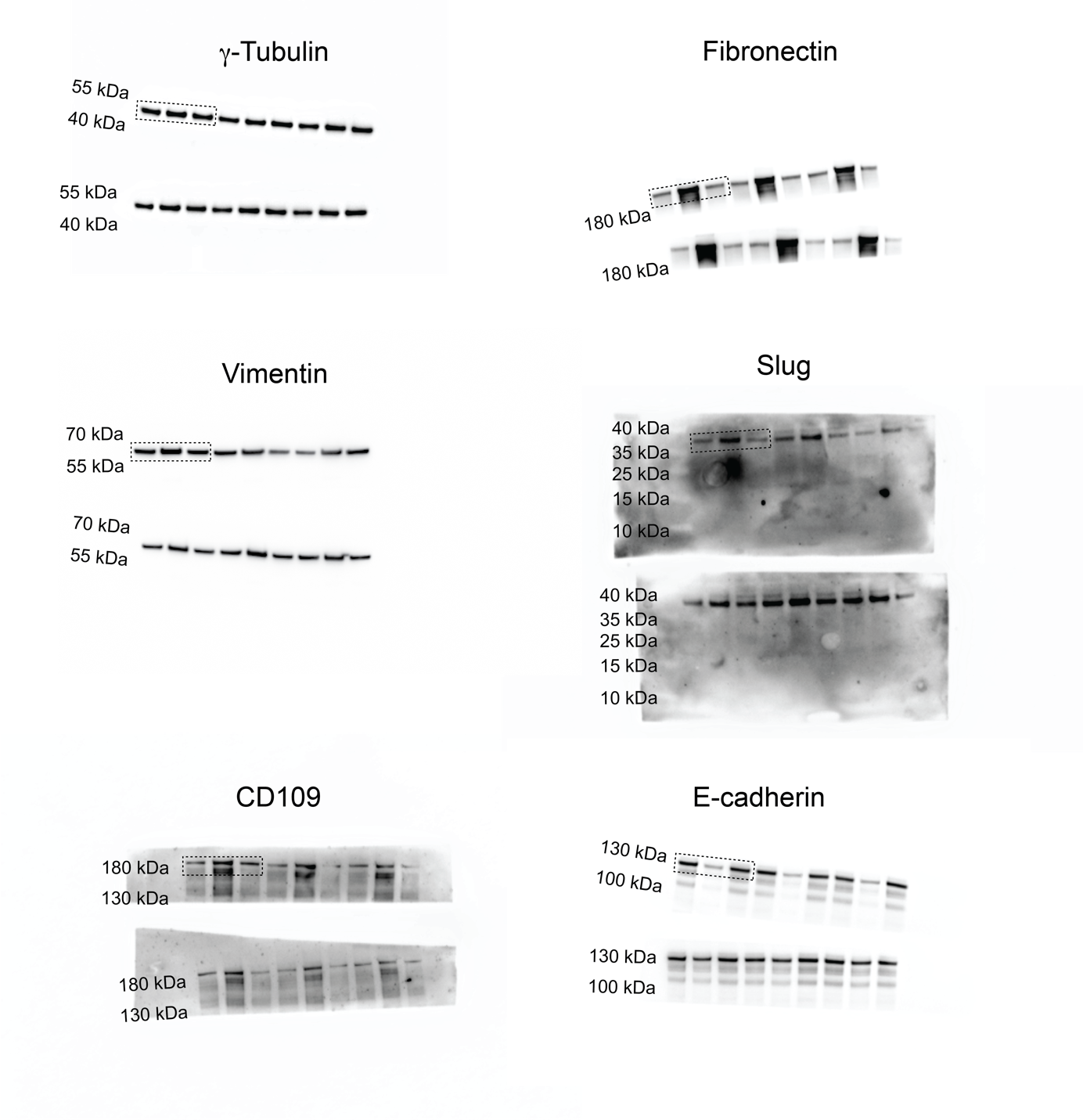
**

**Extended Data Figure 4c**

**
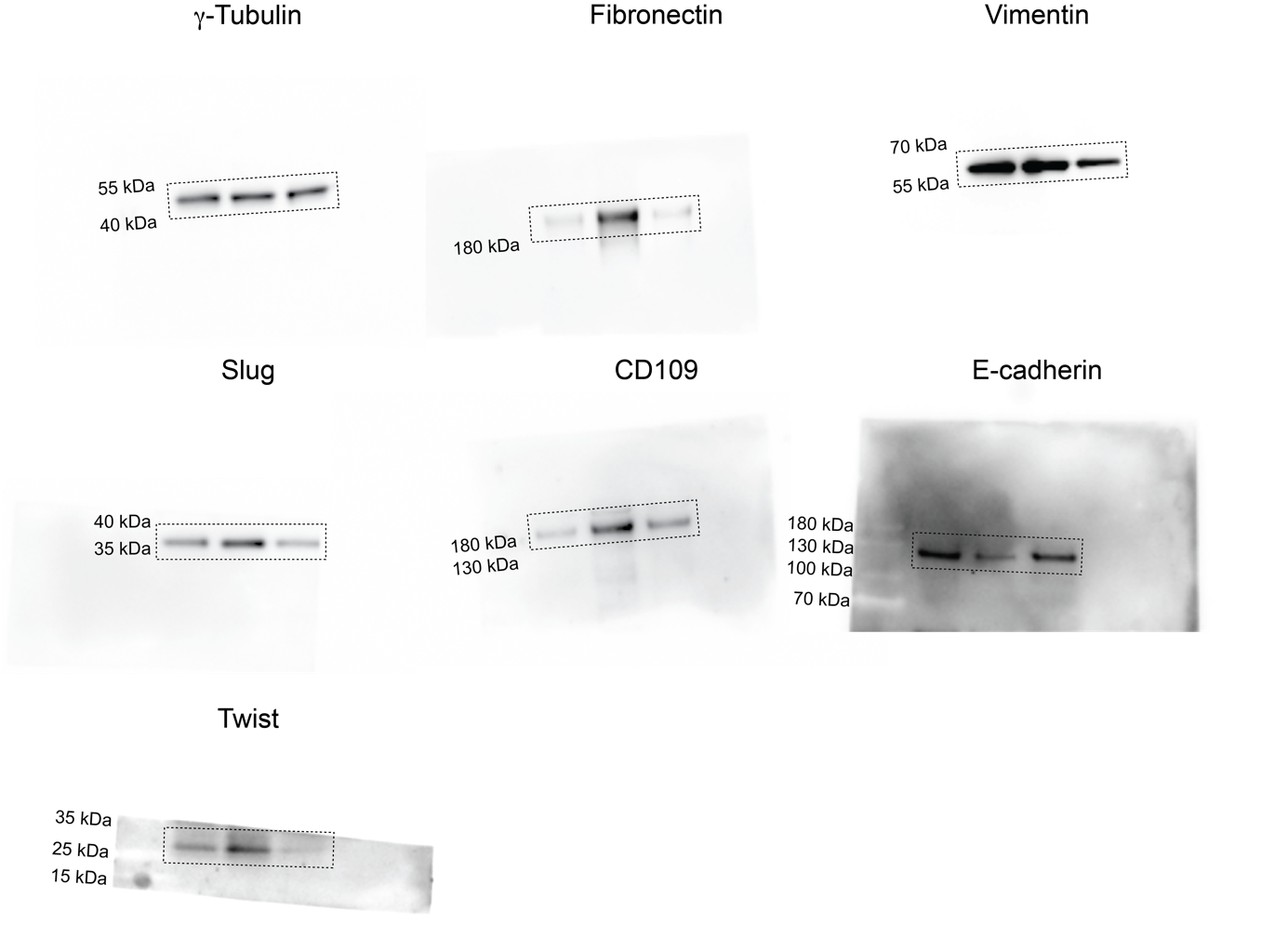
**

**Extended Data Figure 4d**


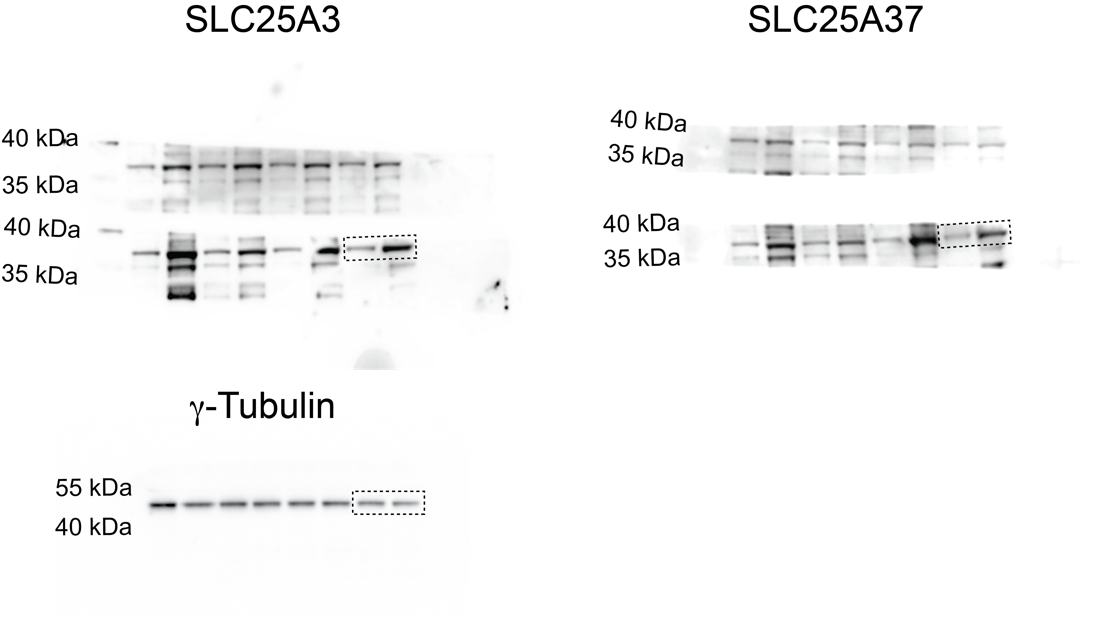


**Extended Data Figure 5k**

**
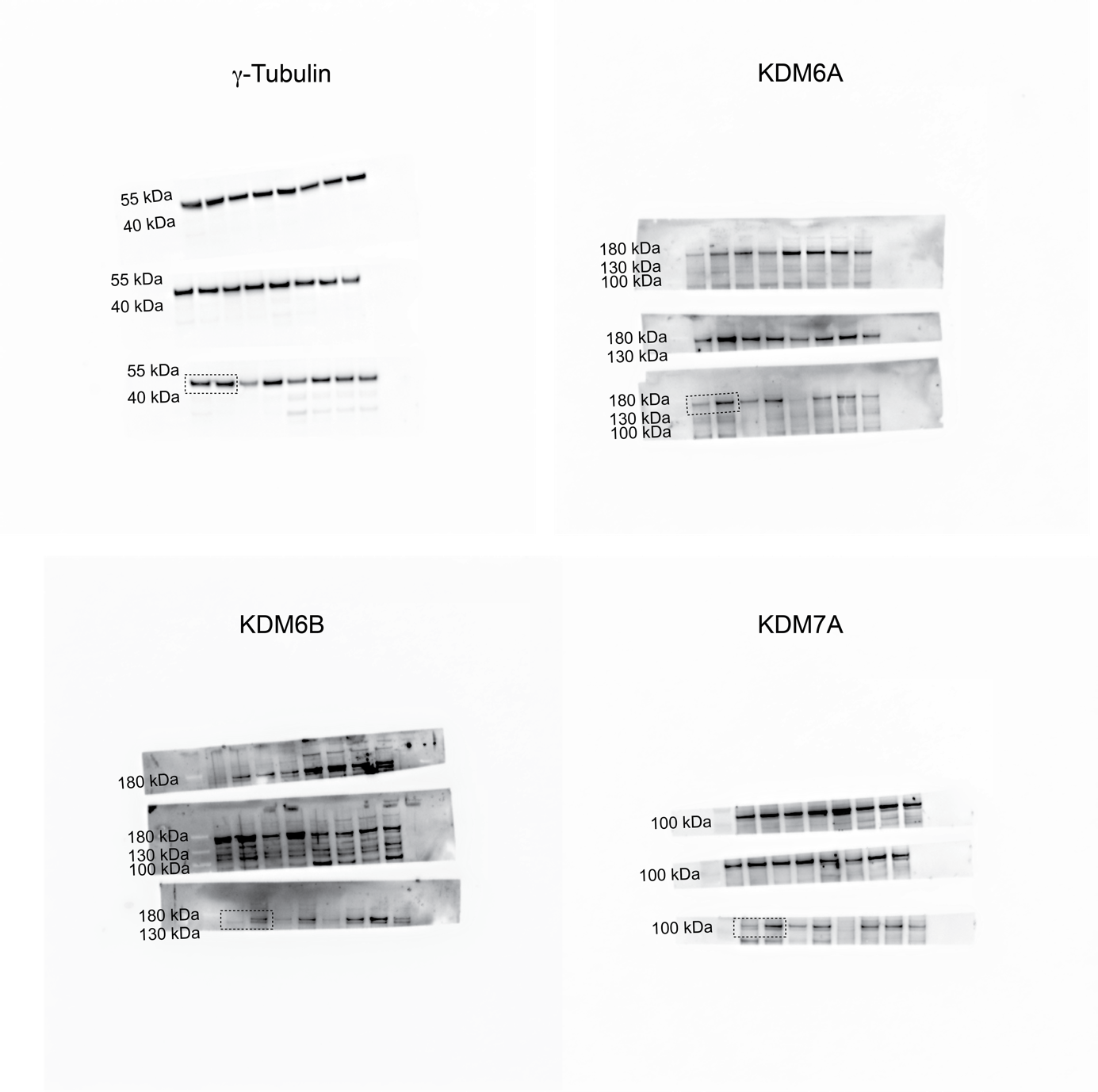
Extended Data Figure 8b**

**
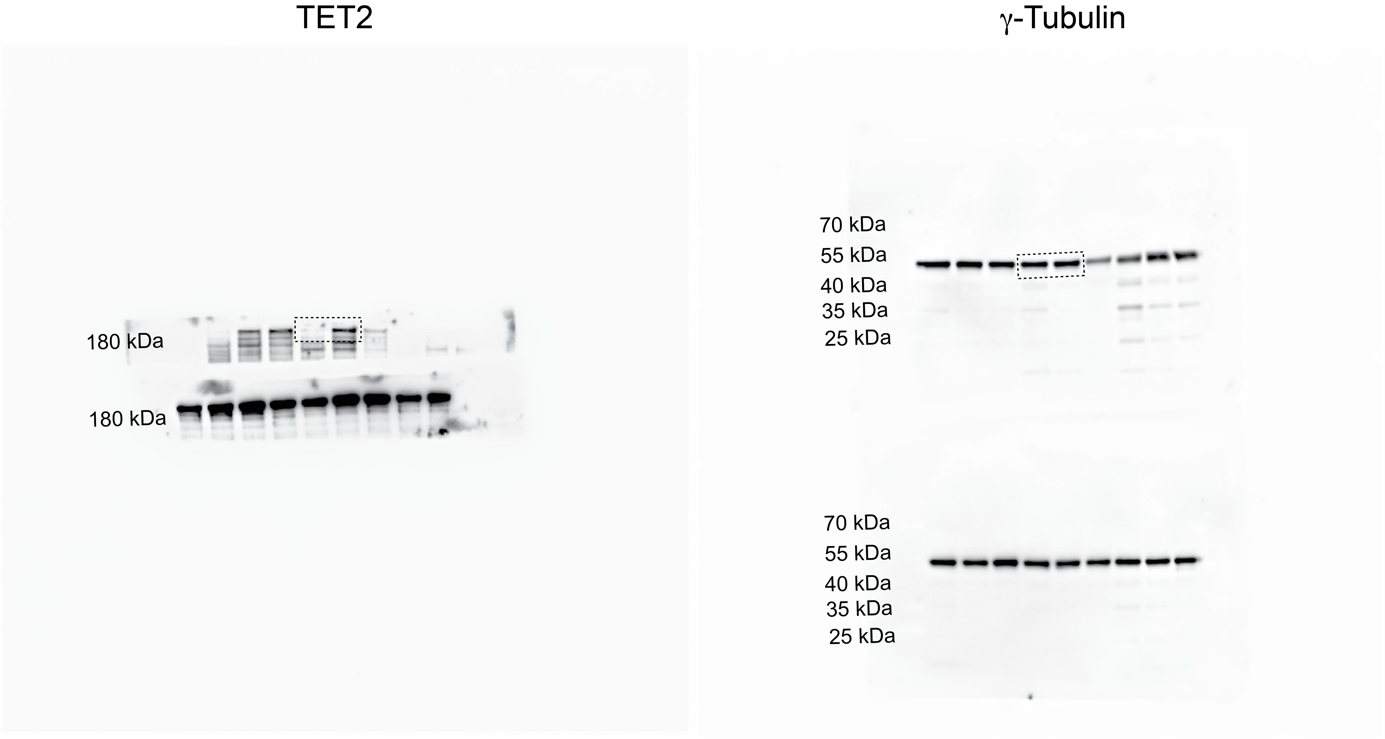
**

**Extended Data Figure 8b – continued**

**
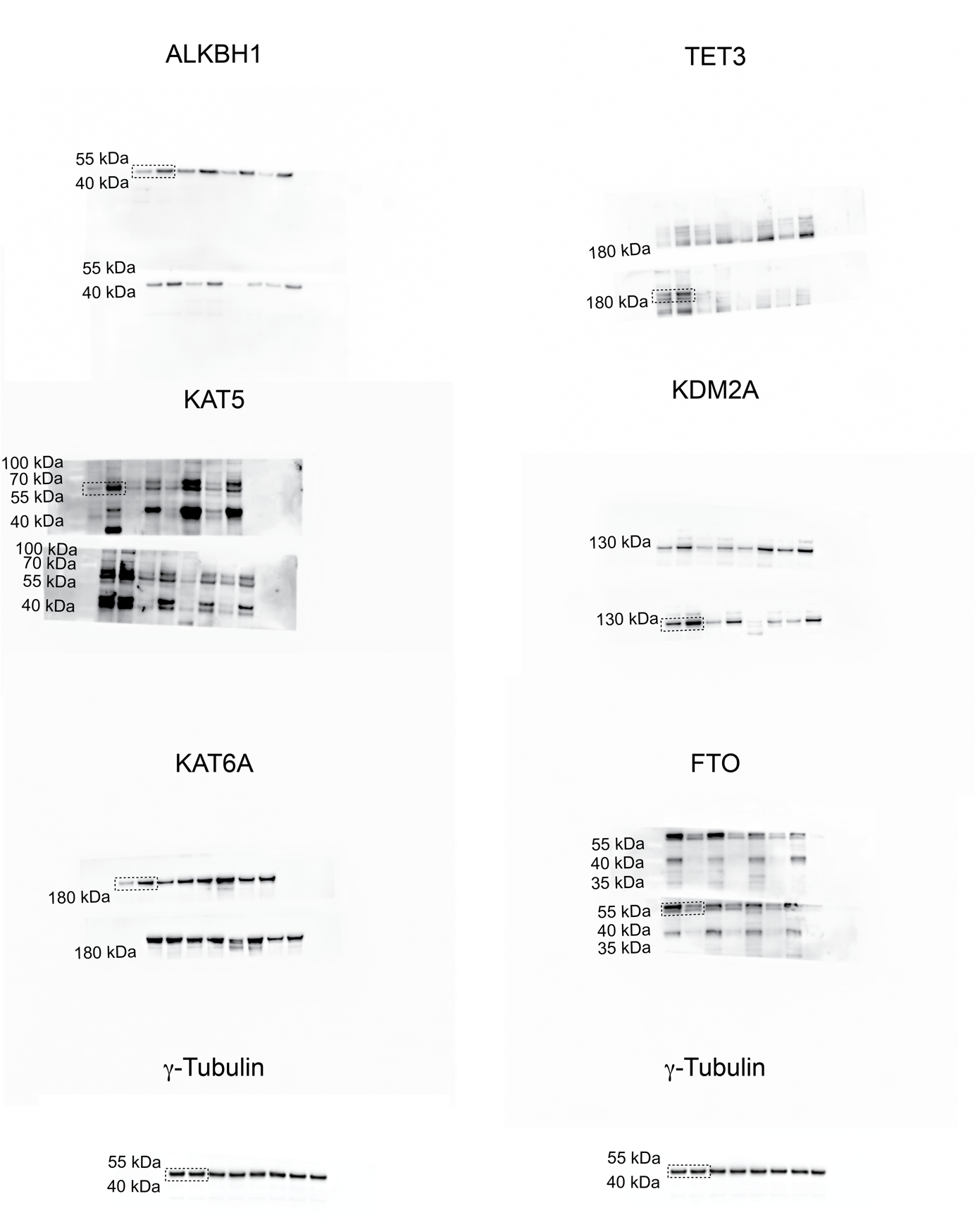
**

**Extended Data Figure 8b – continued**

**
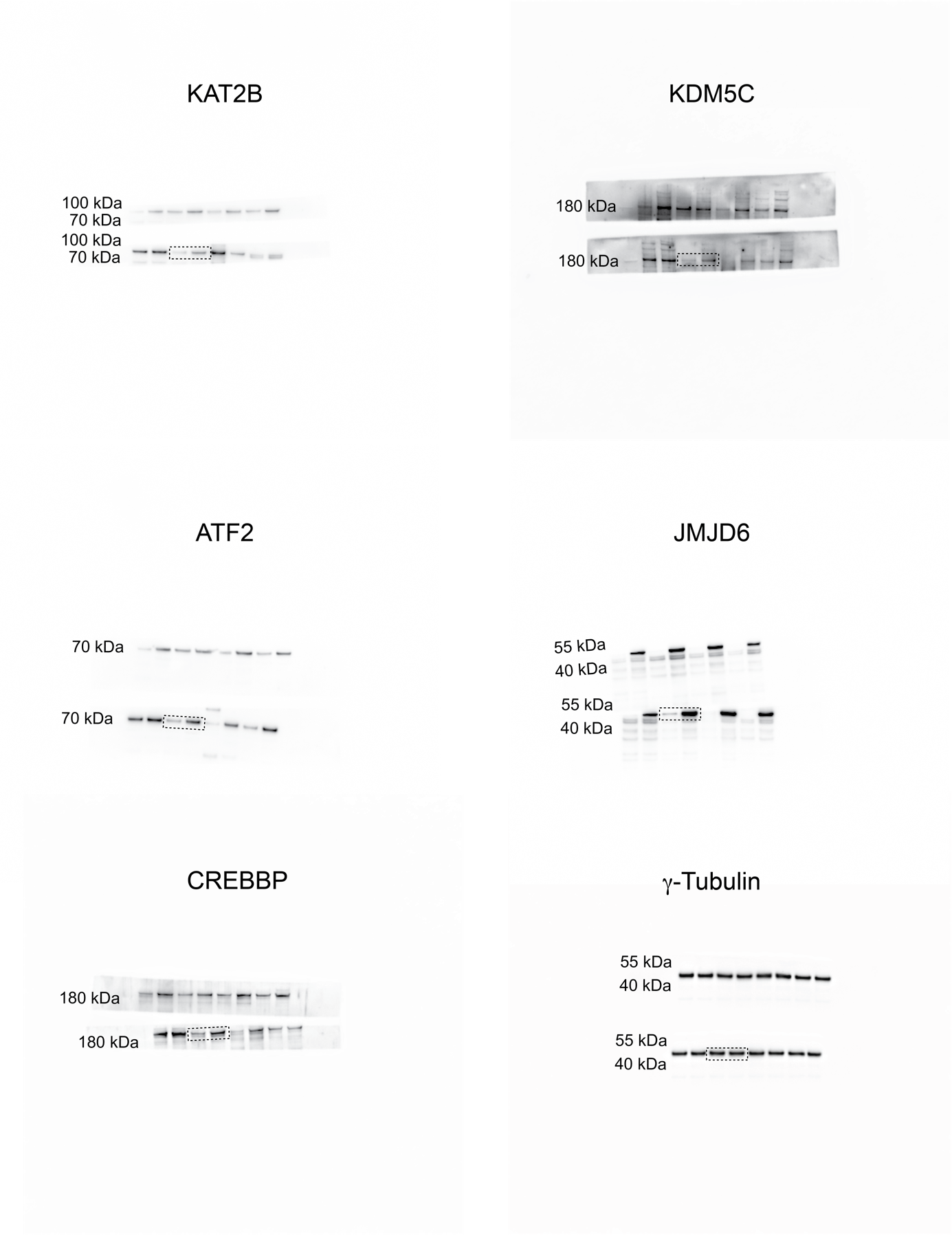
**

**Extended Data Figure 8b – continued**

**
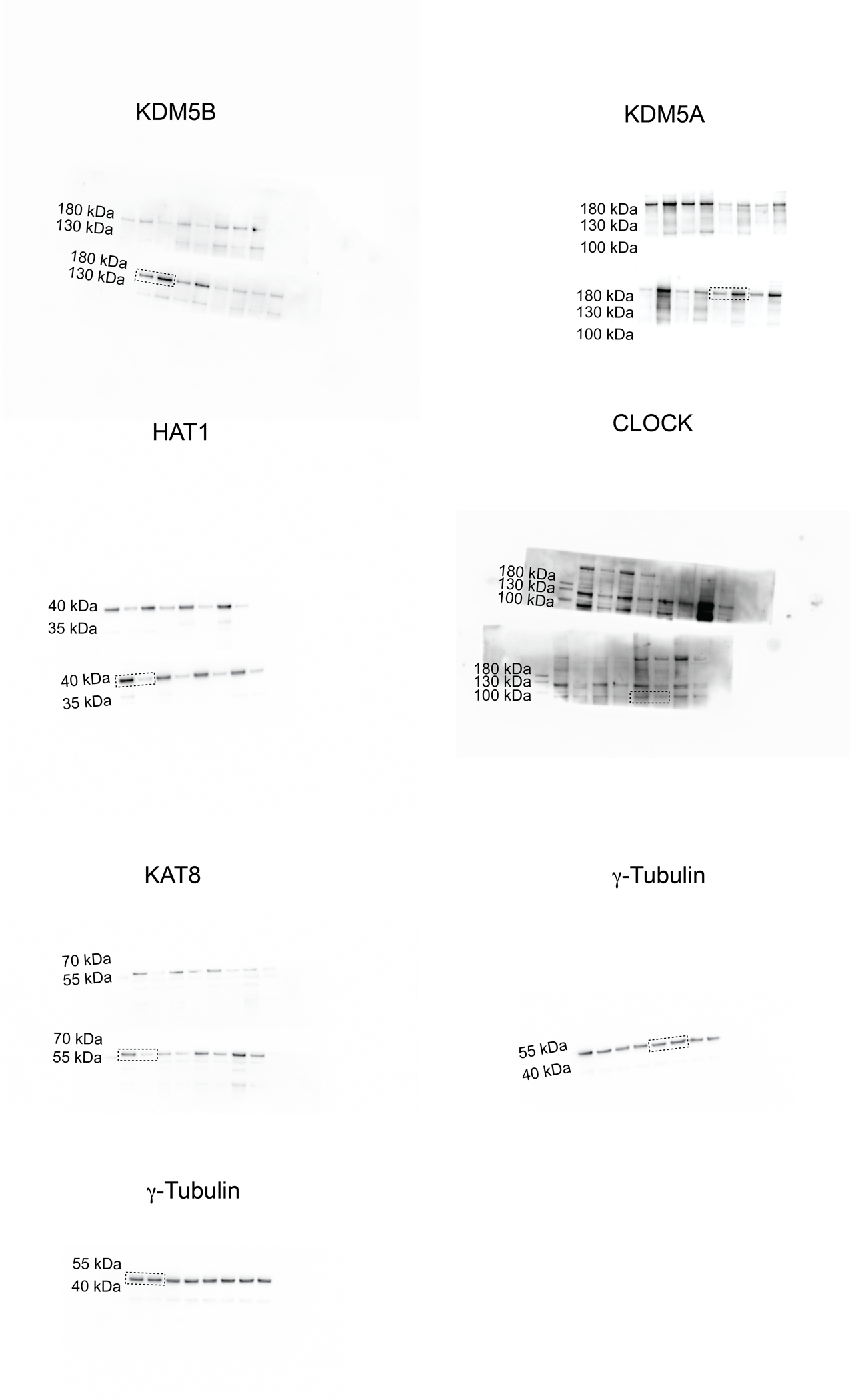
**

**Extended Data Figure 8b – continued**

**
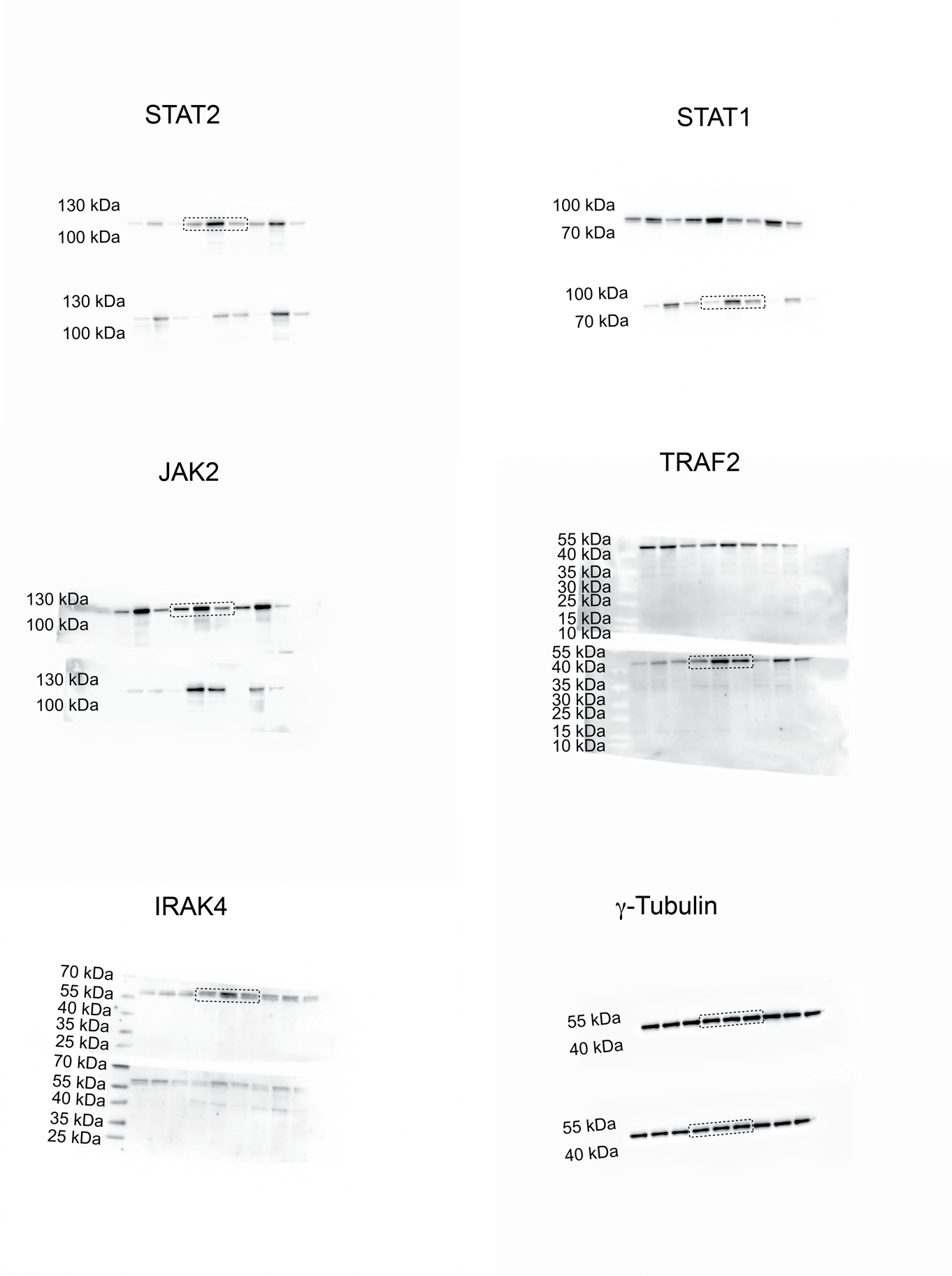
**

**Extended Data Figure 9a**

**
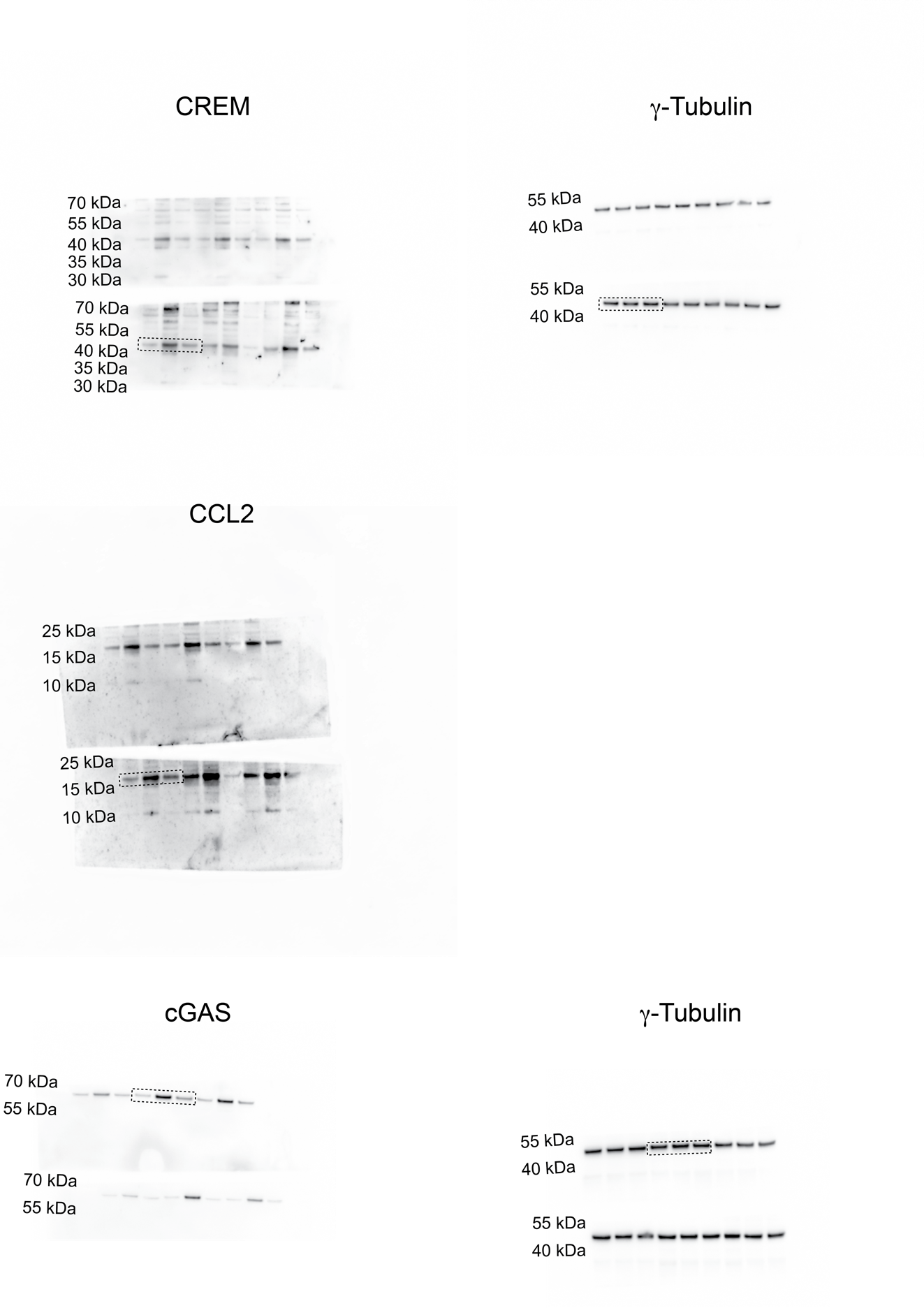
**

**Extended Data Figure 9a - continued**

**
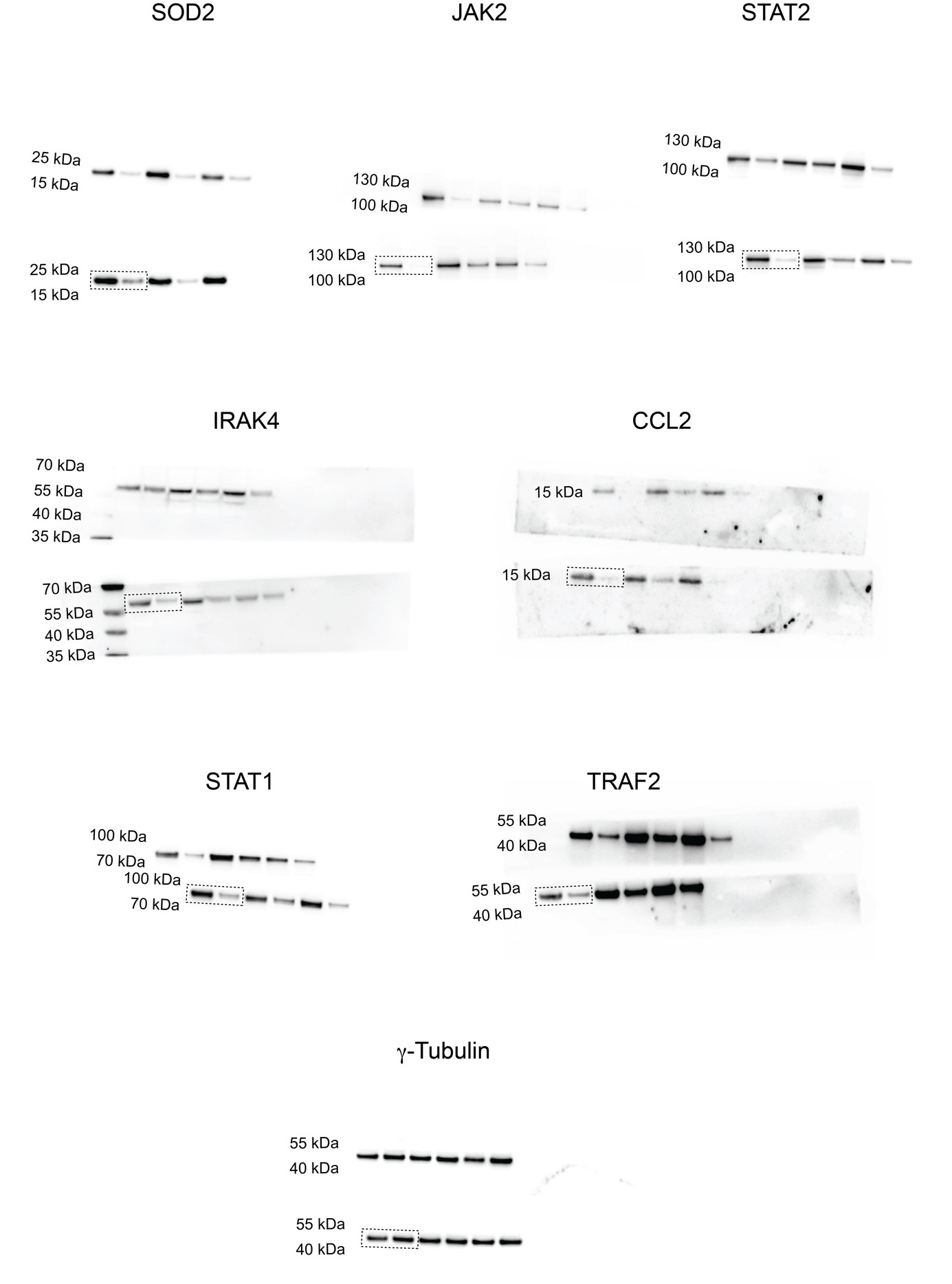
**

**Extended Data Figure 9g**

**
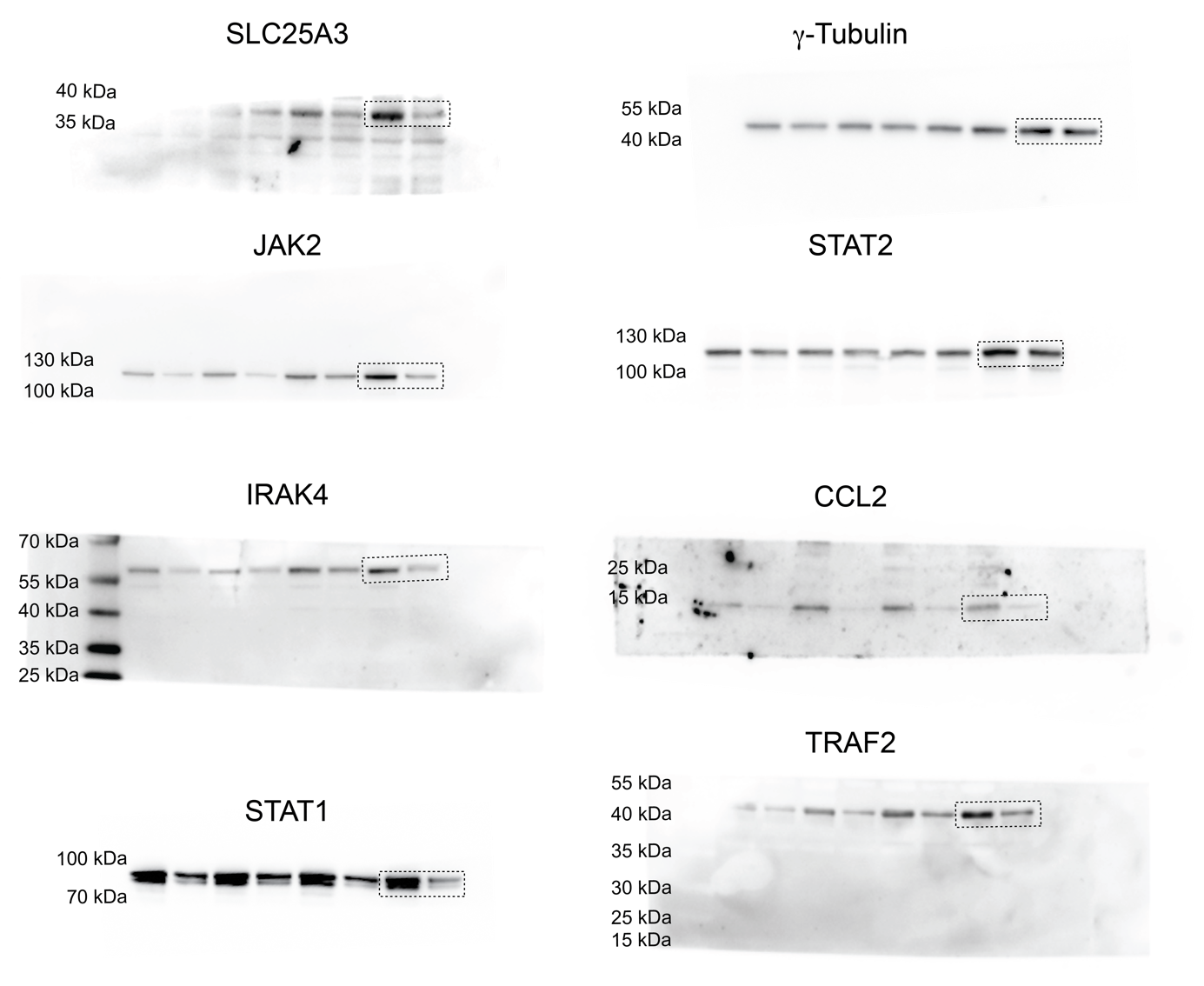
**

**Extended Data Figure 9h**

**
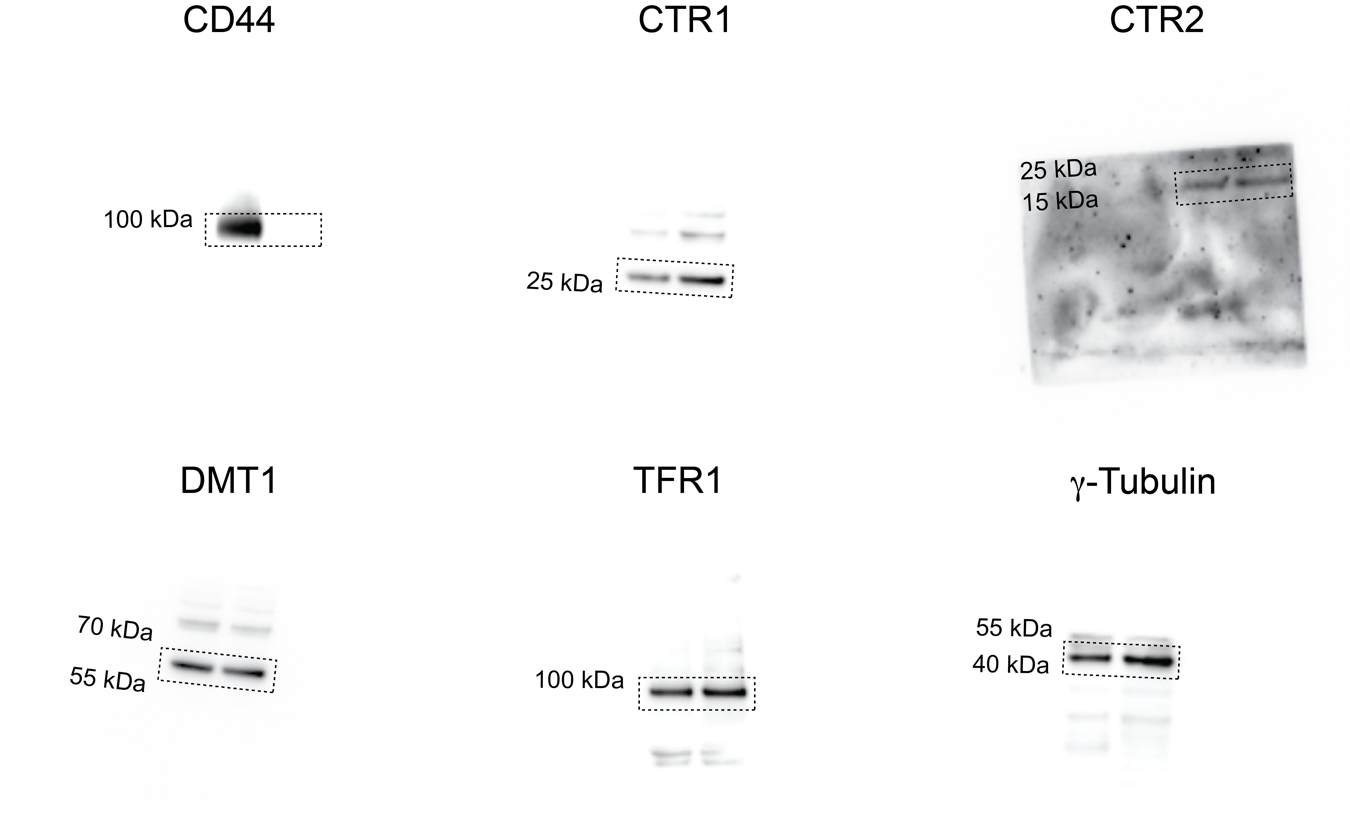
**

**Extended Data Figure 9j**

**
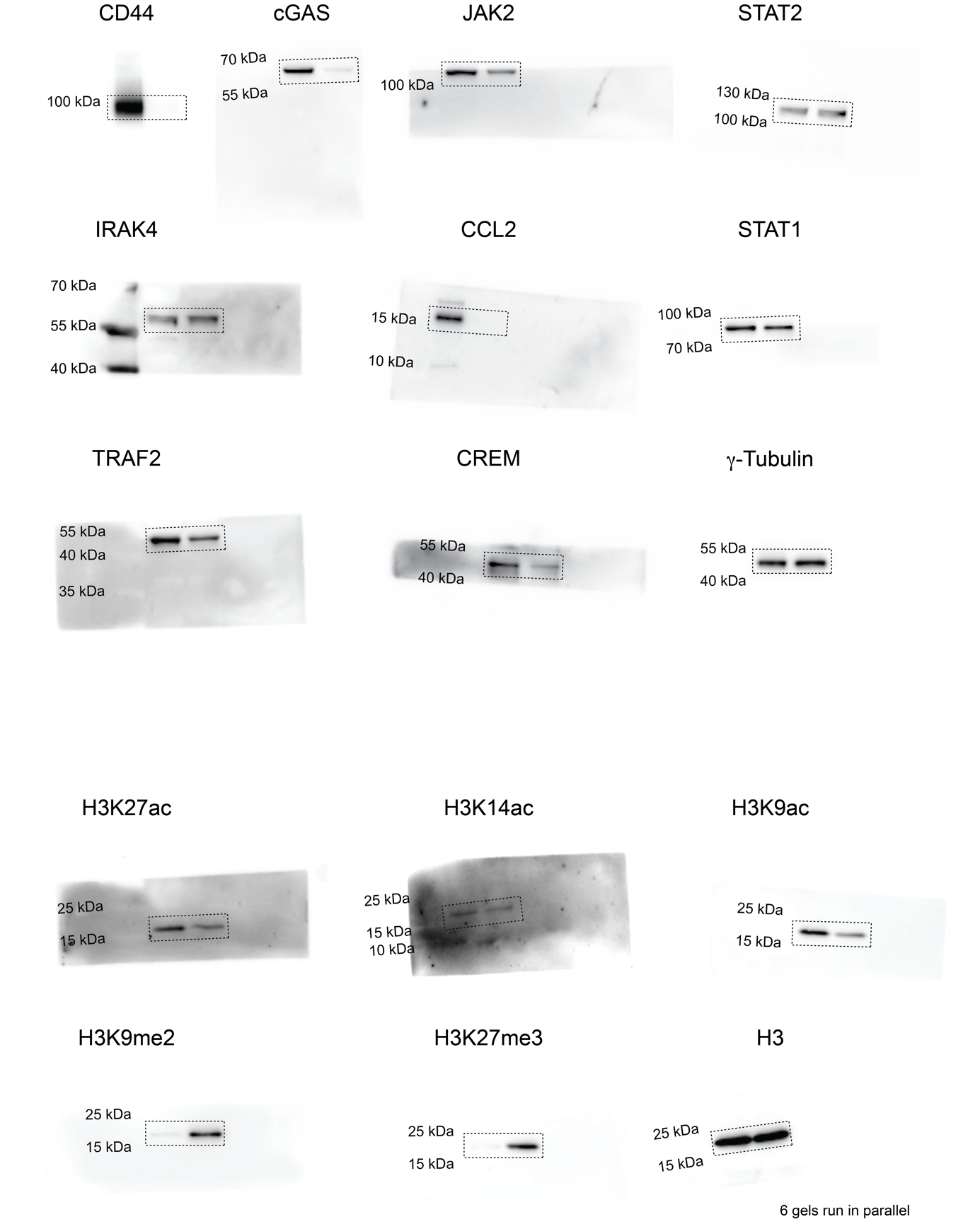
**

**Extended Data Figure 9k (Donor 1)**

**
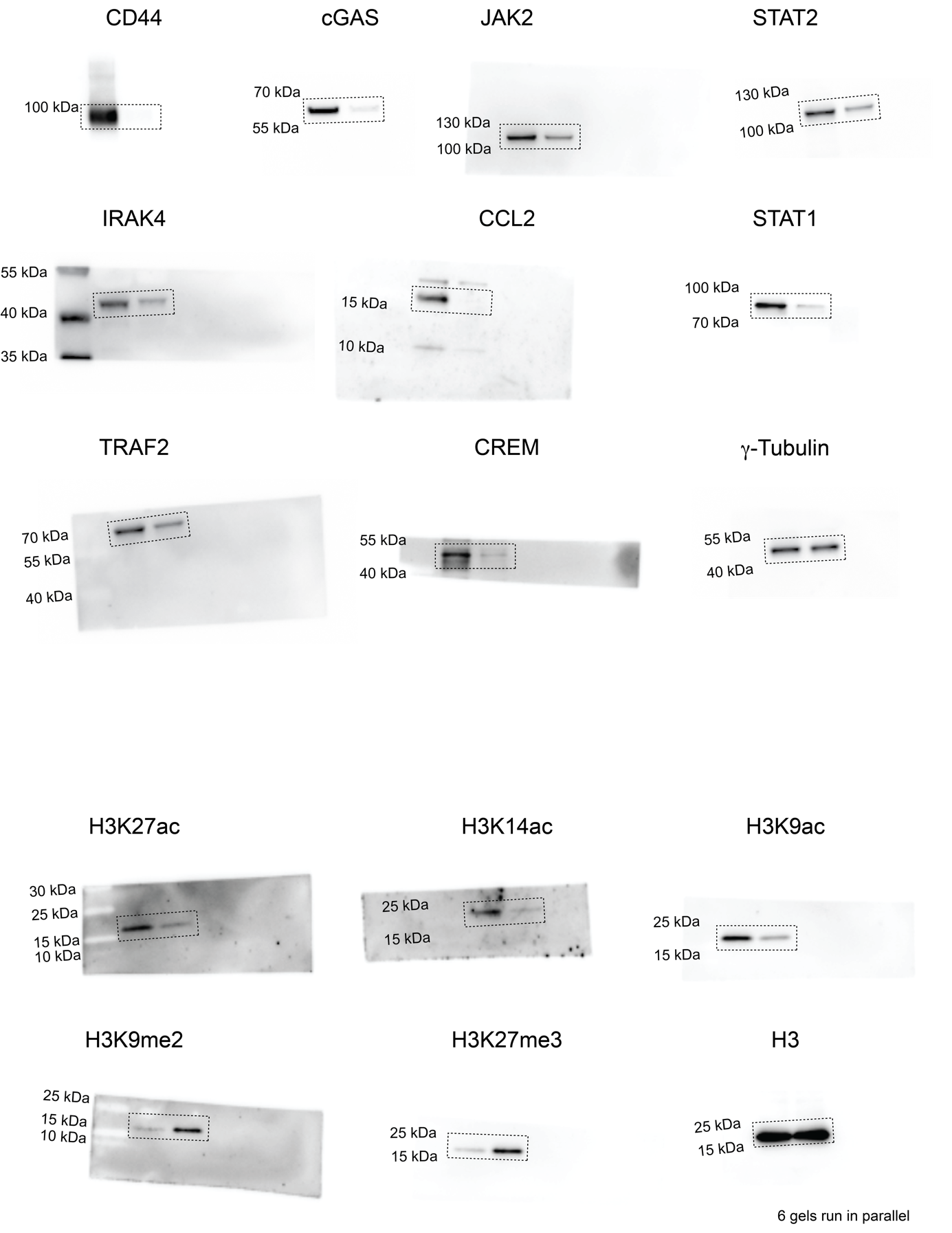
**

**Extended Data Figure 9k (Donor 2)**

**
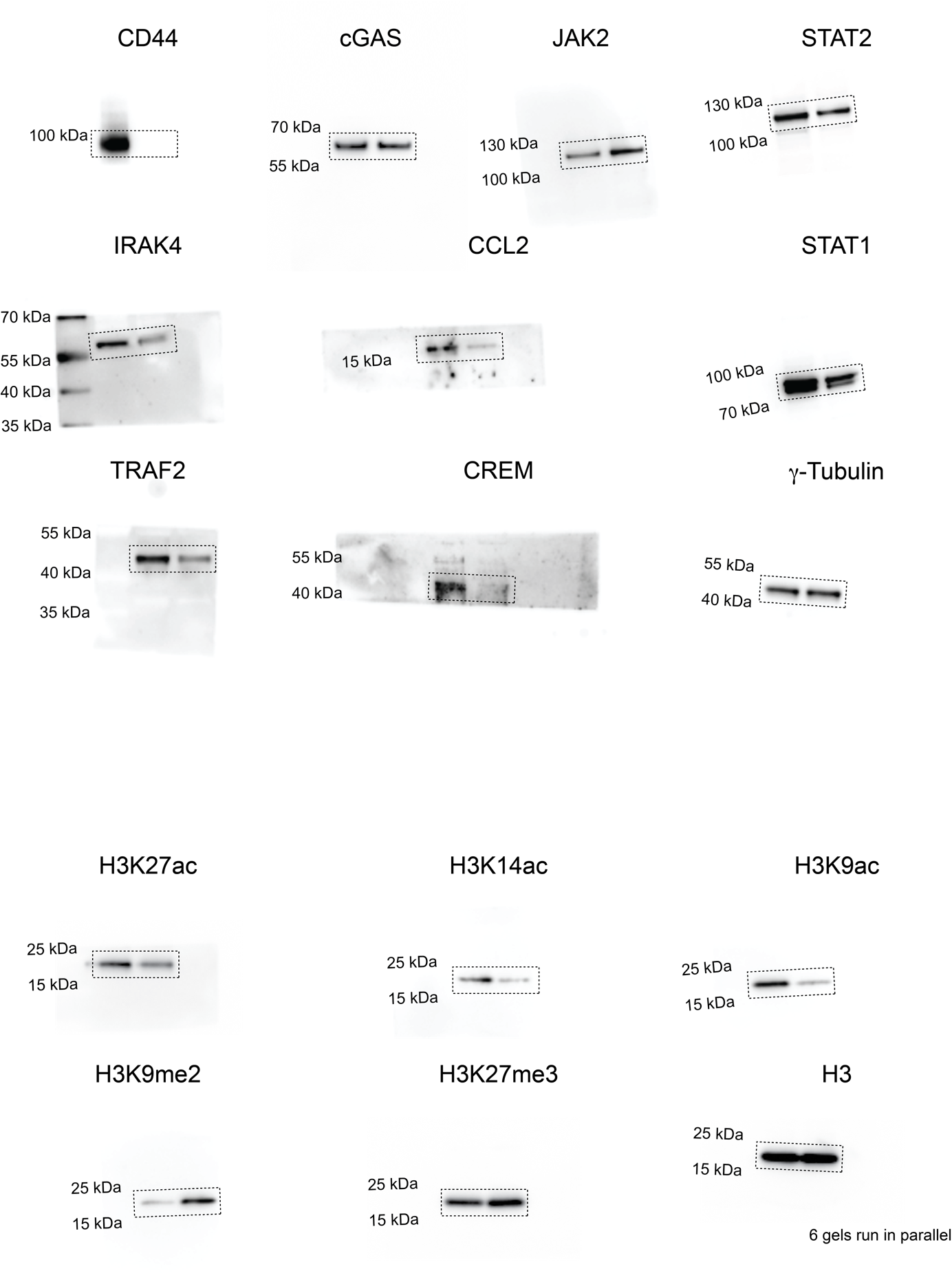
**

**Extended Data Figure 9k (Donor 3)**

**
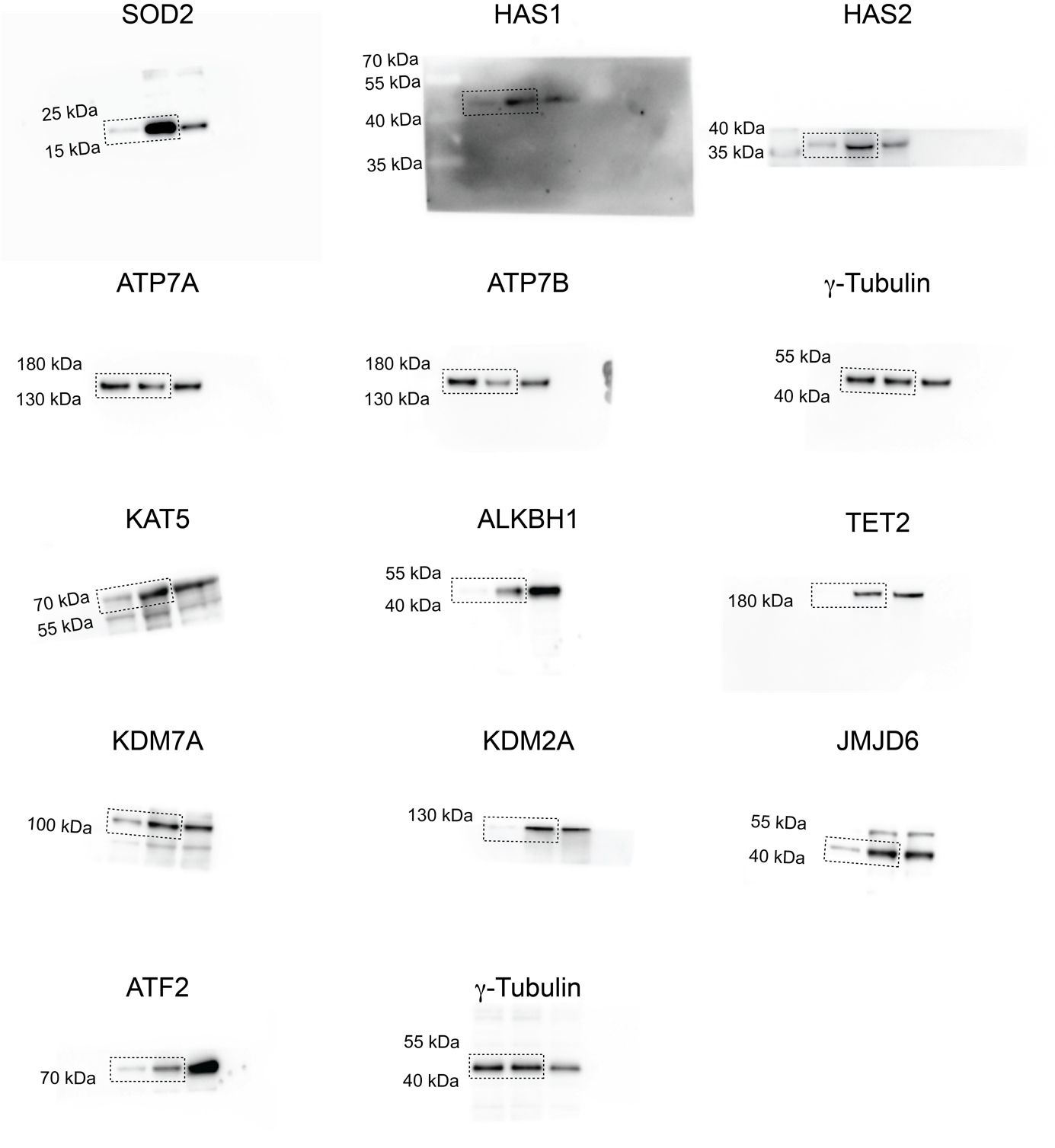
**

**Extended Data Figure 10a**

**
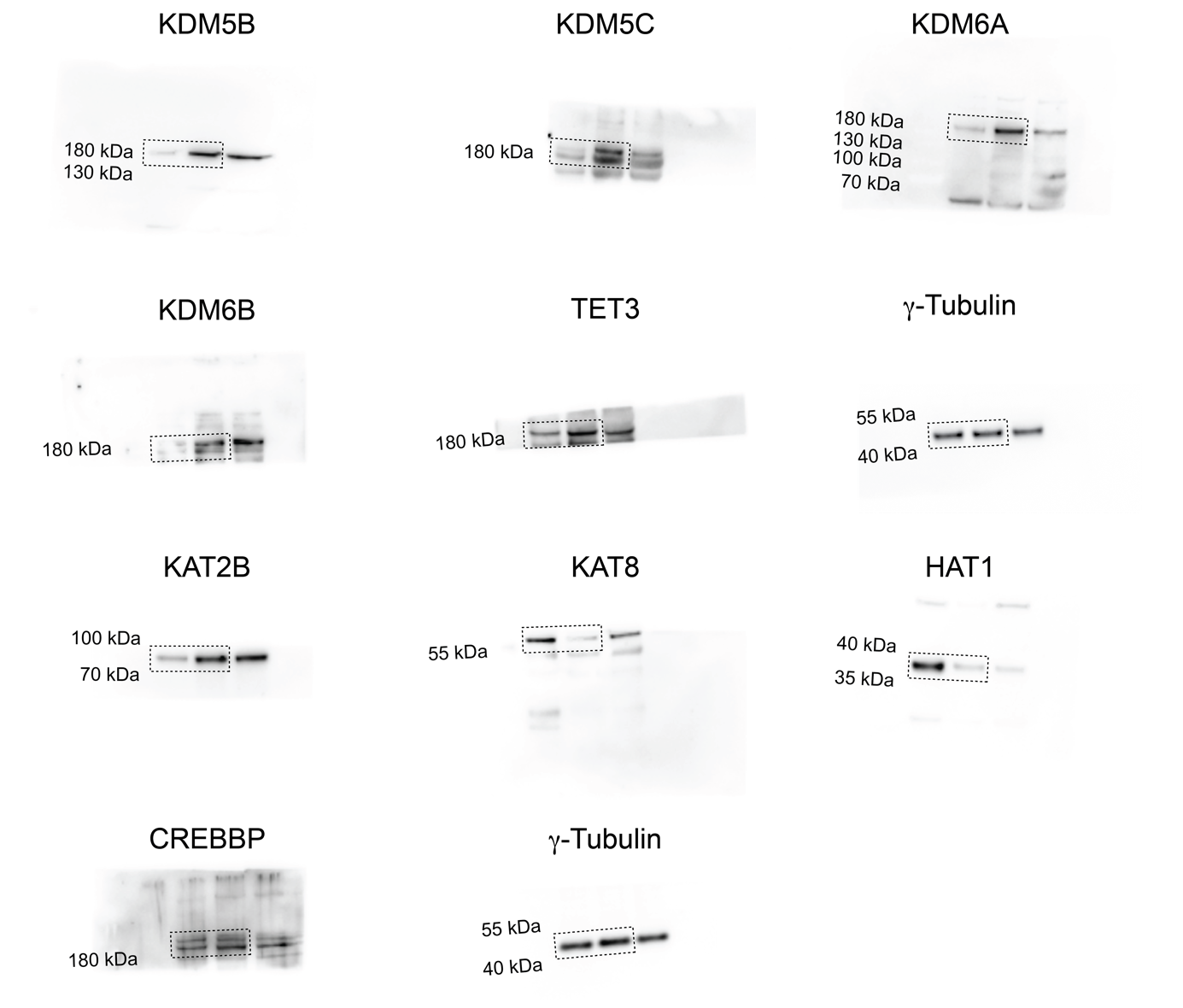
**

**Extended Data Figure 10a – continued**

**
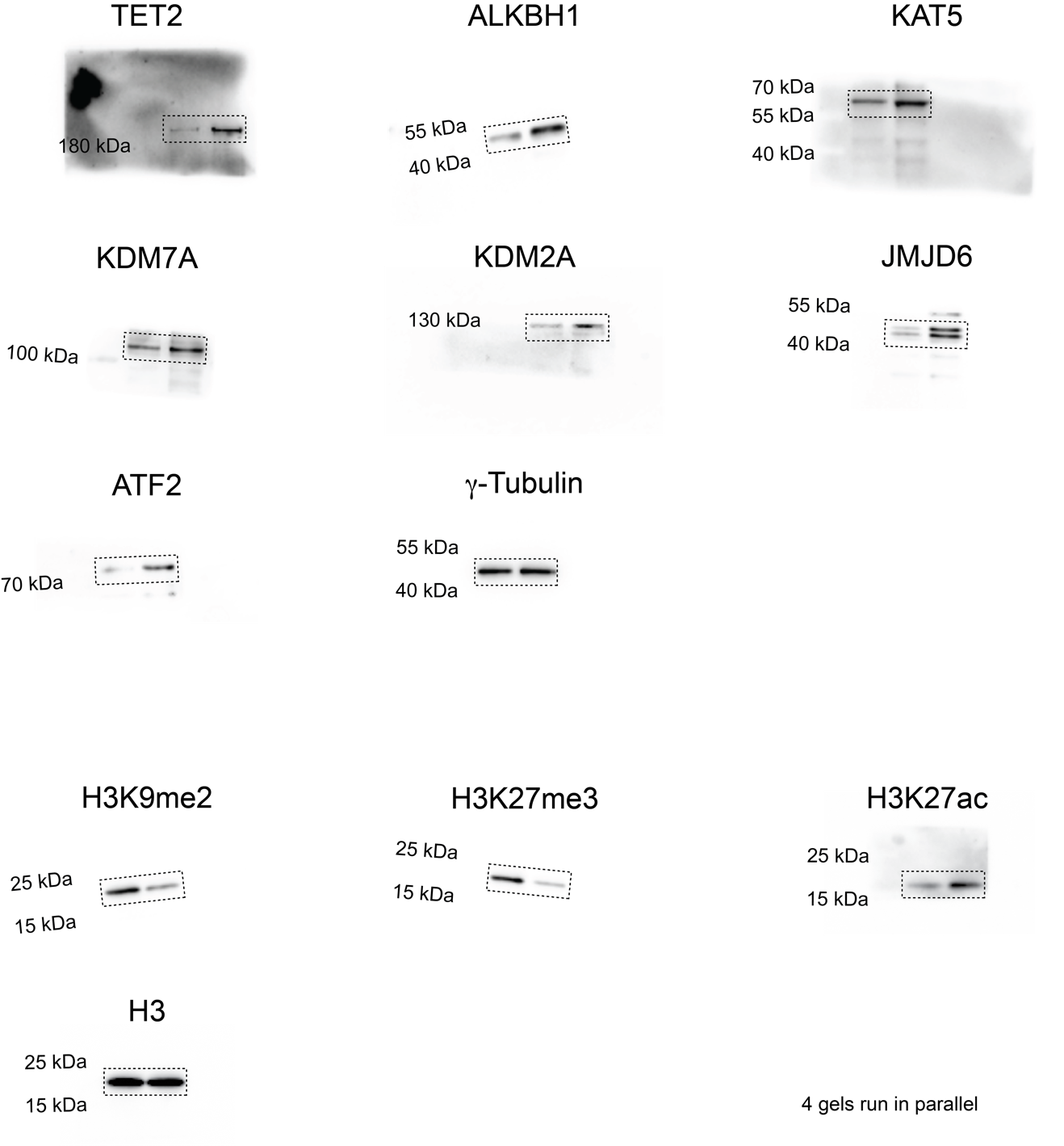
**

**Extended Data Figure 10b**

**_
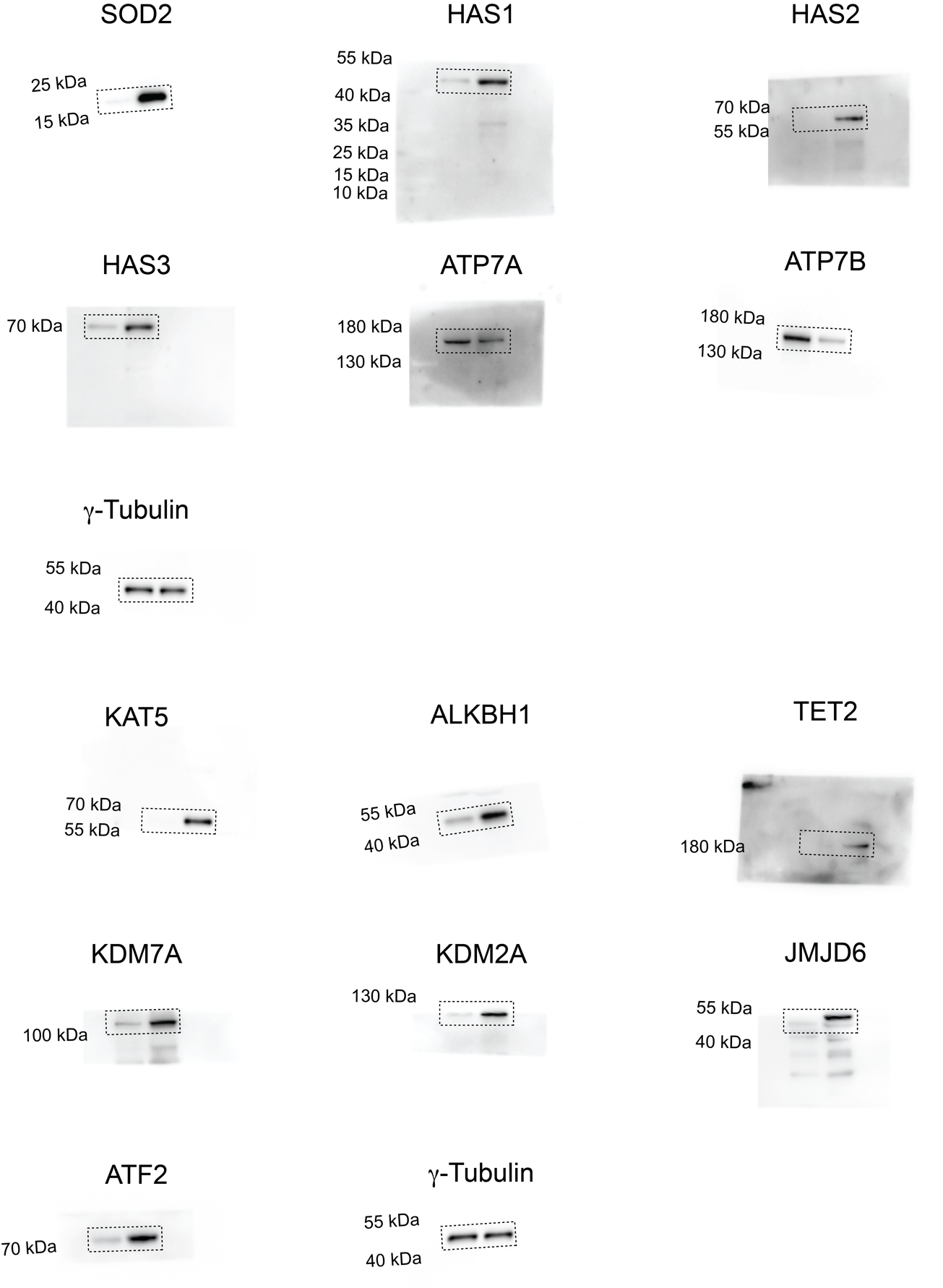
_**

**Extended Data Figure 10c**

**
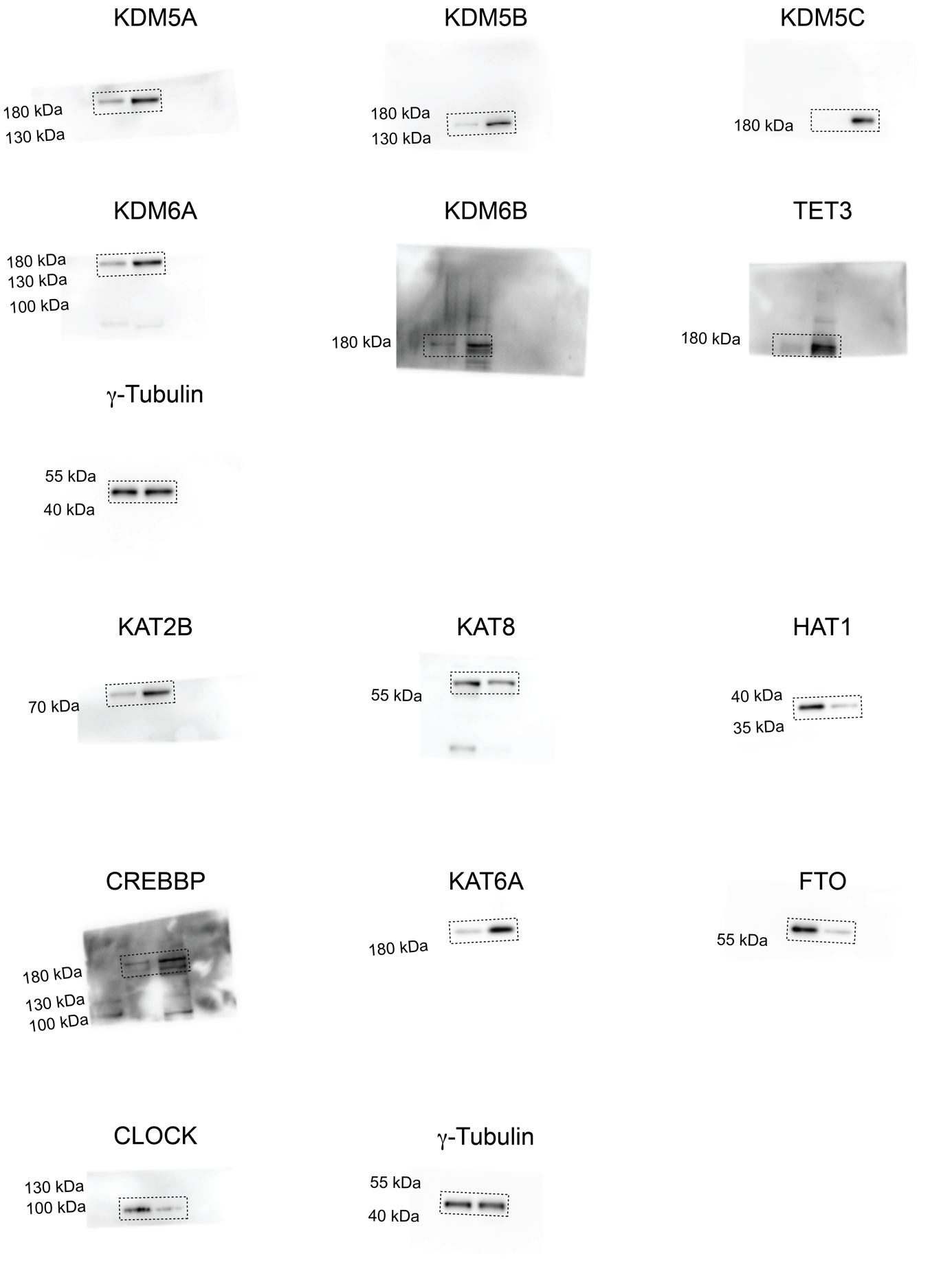
**

**Extended Data Figure 10c – continued**

**
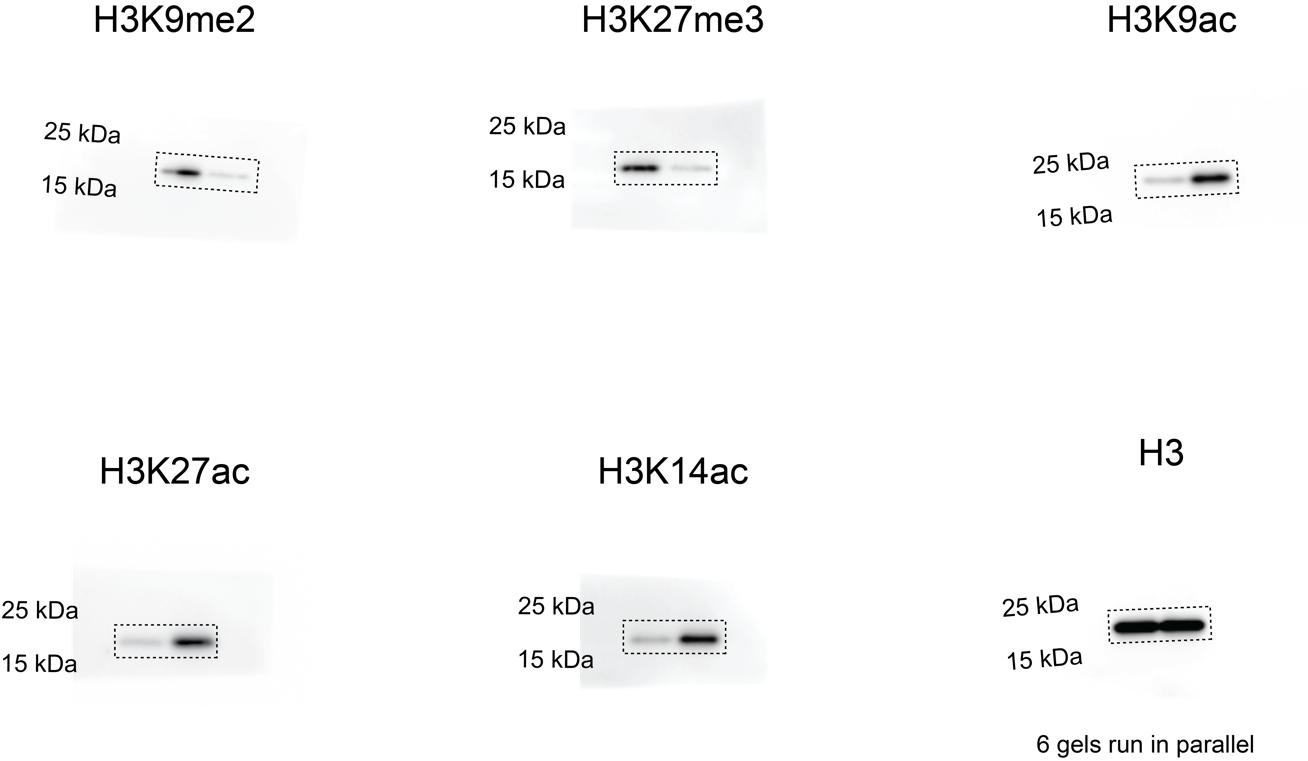
**

**Extended Data Figure 10c – continued**

**^1^H NMR spectrum of LCC-12 in DMSO-*d_6_***


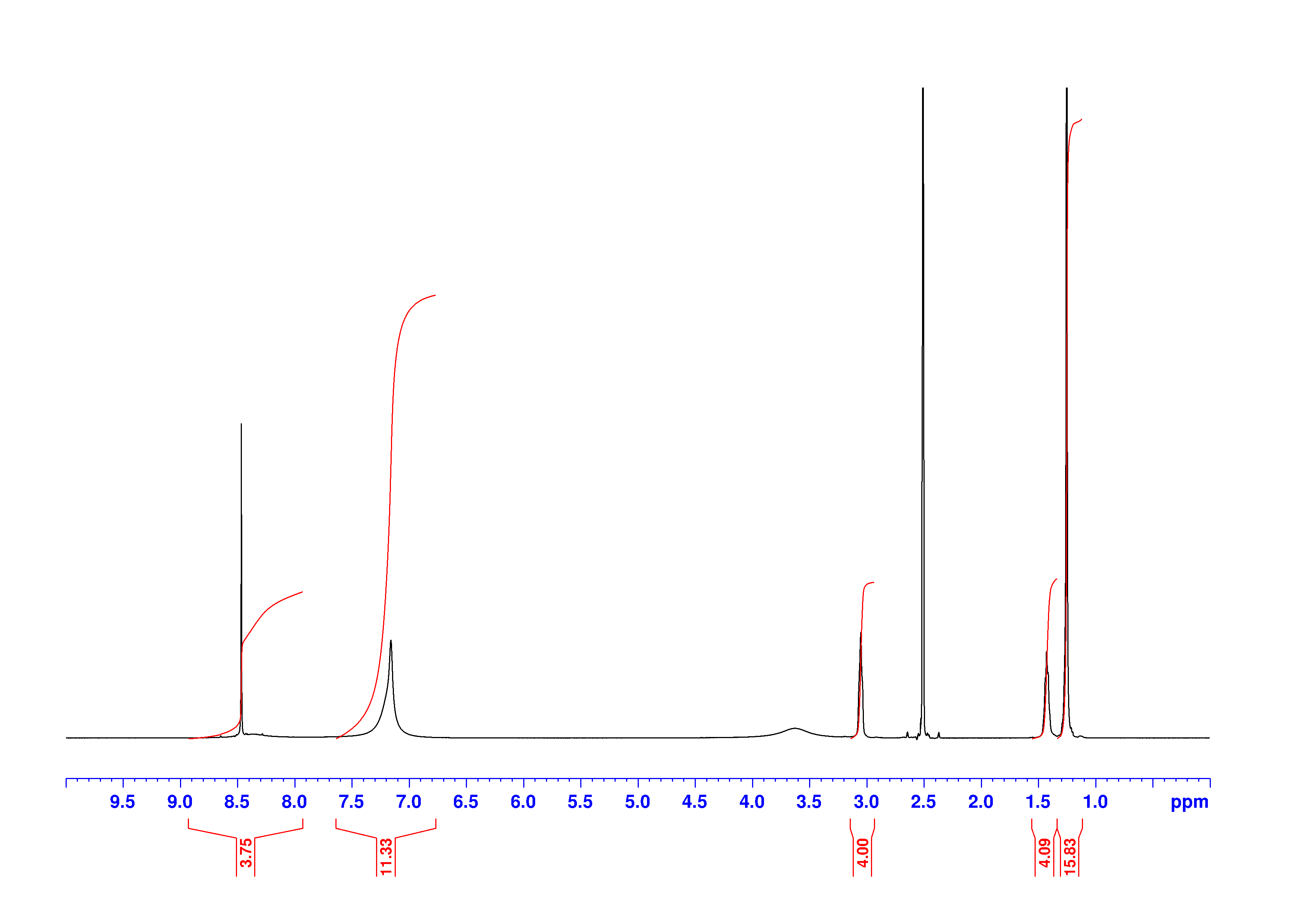


**^13^C NMR spectrum of LCC-12 in DMSO-*d_6_***


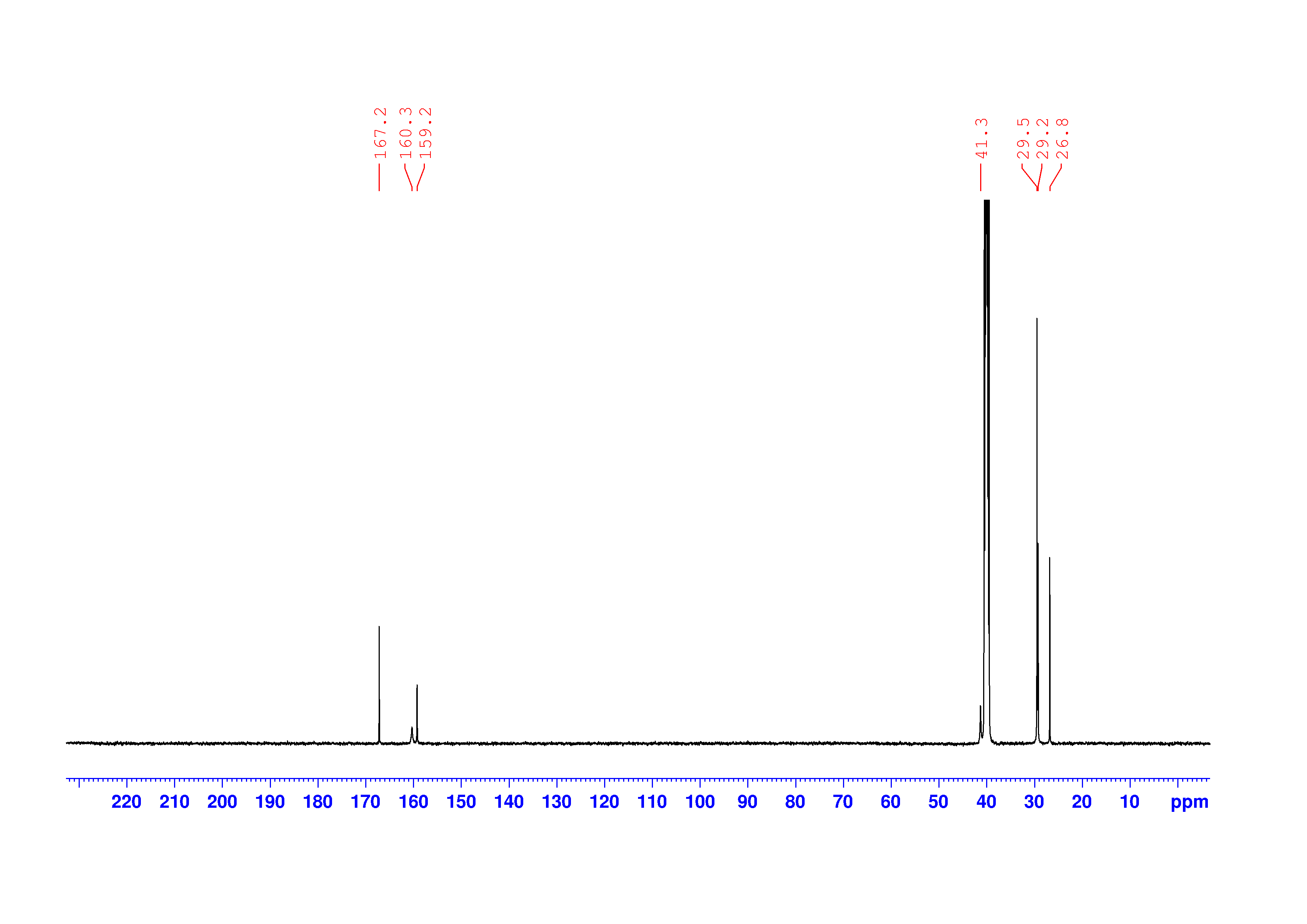


**^1^H NMR spectrum of LCC-4,4 in DMSO-*d_6_***


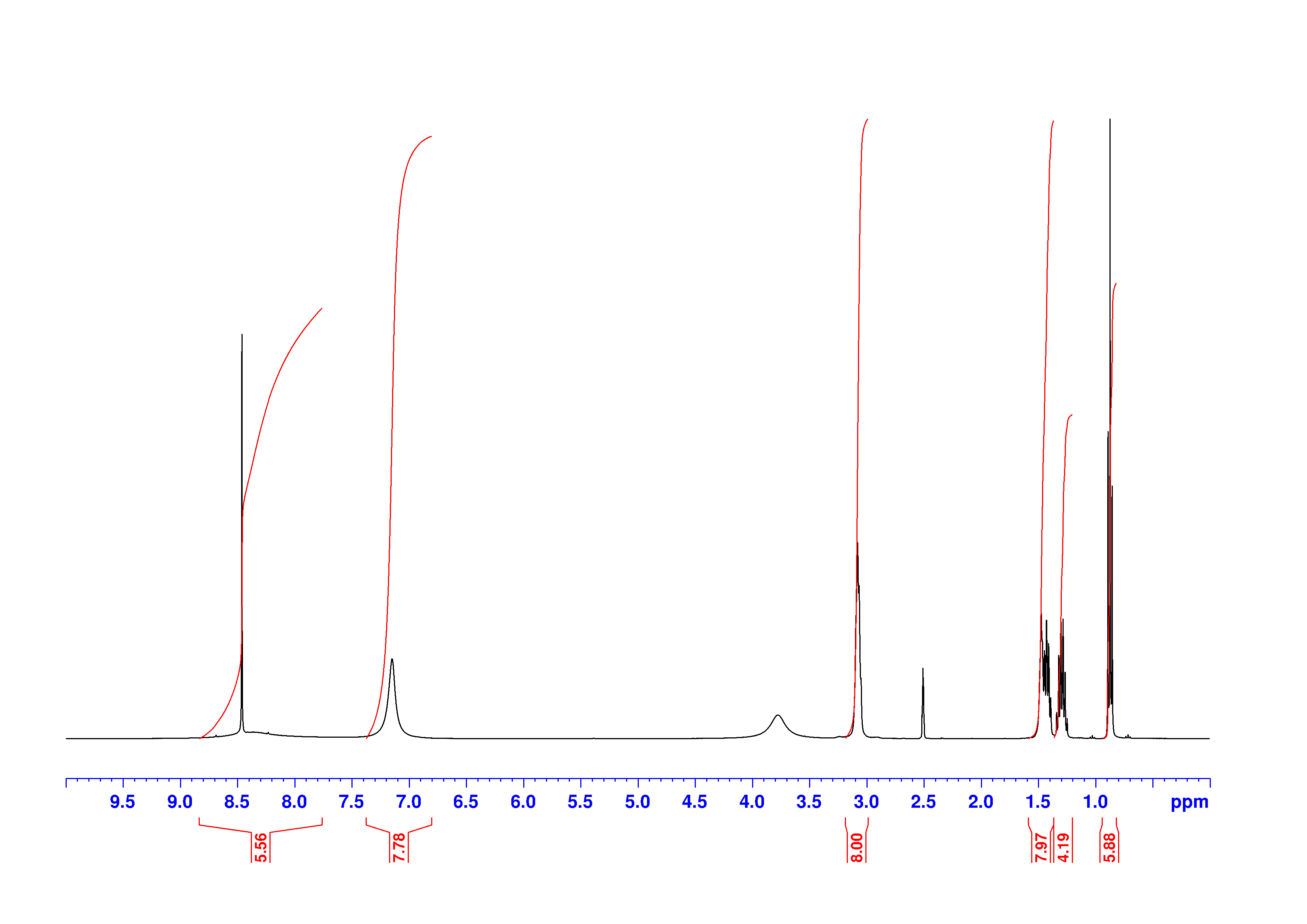


**^13^C NMR spectrum of LCC-4,4 in DMSO-*d_6_***


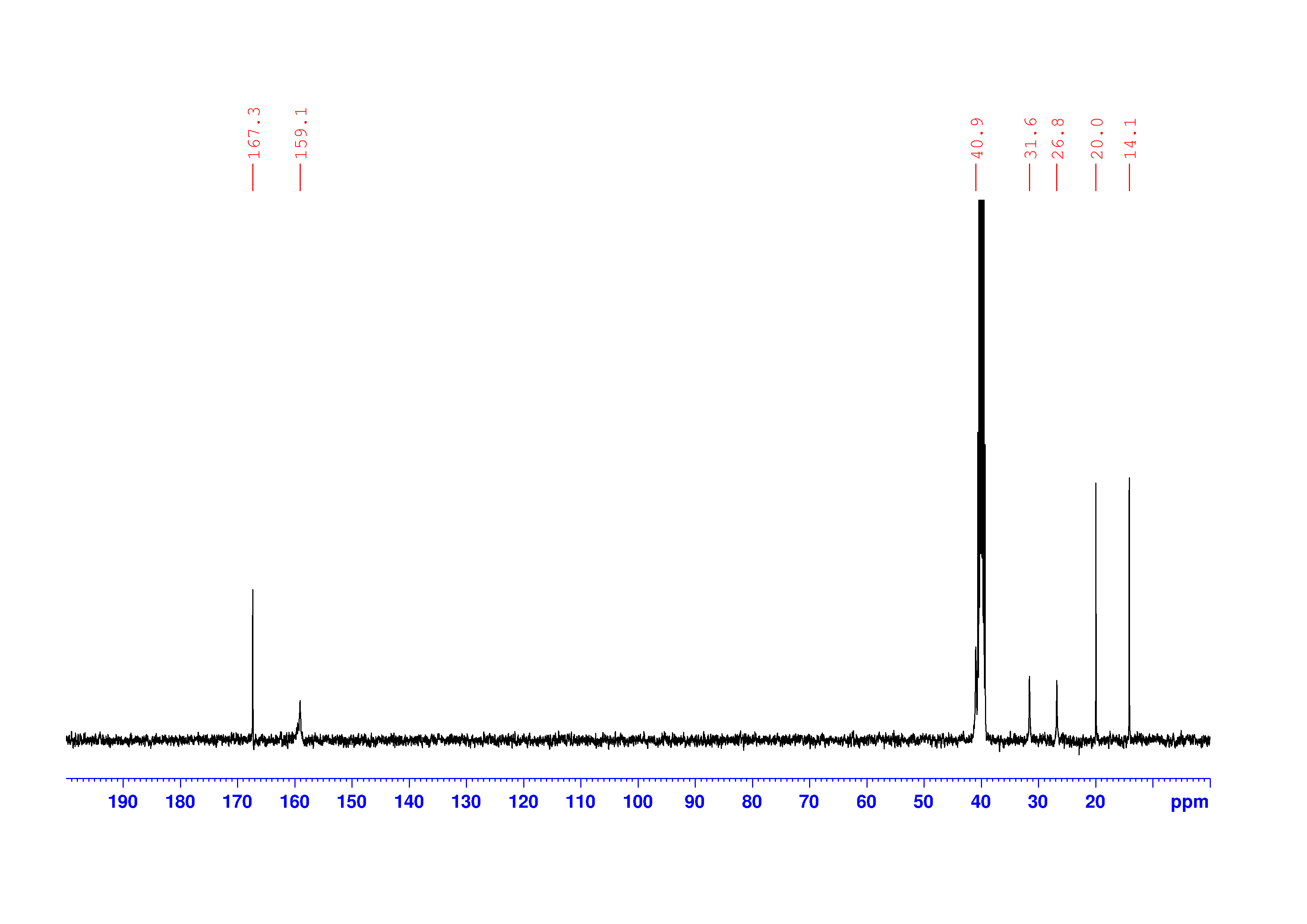


**^1^H NMR spectrum of LCC-12,4 in DMSO-*d_6_***


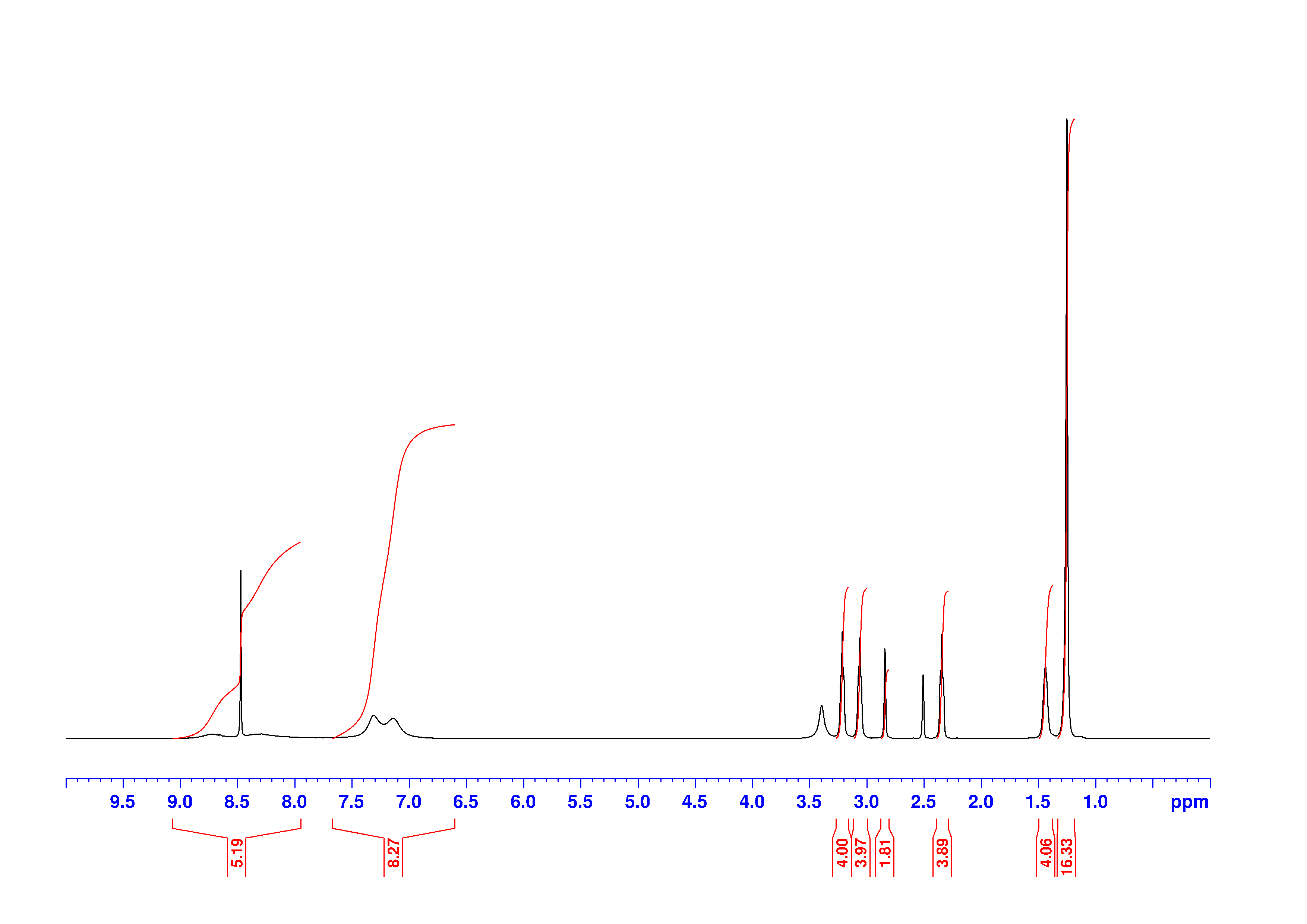


**^13^C NMR spectrum of LCC-12,4 in DMSO-*d_6_***


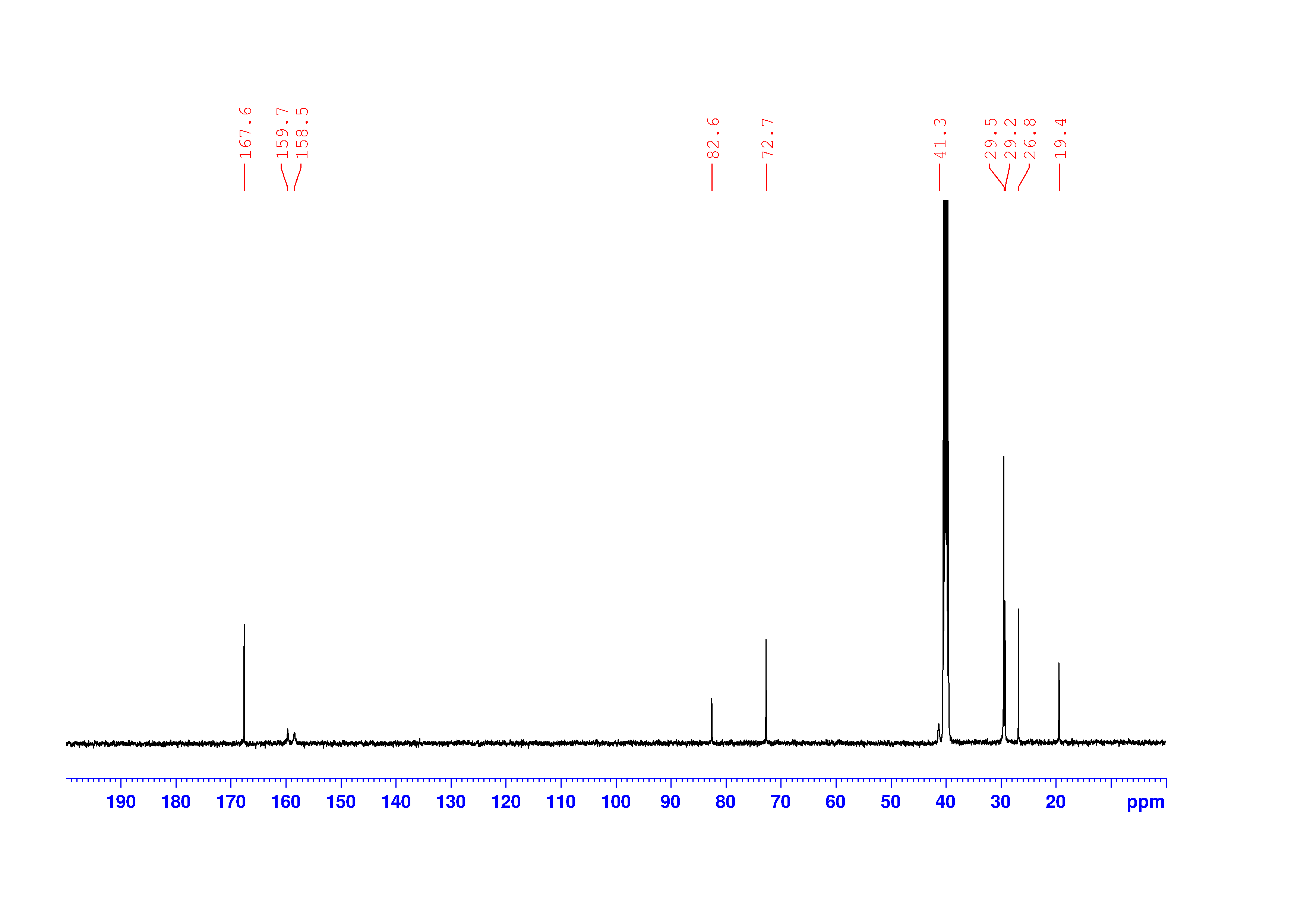


**^1^H NMR spectrum of isotopically labelled LCC-12 in DMSO-*d_6_***


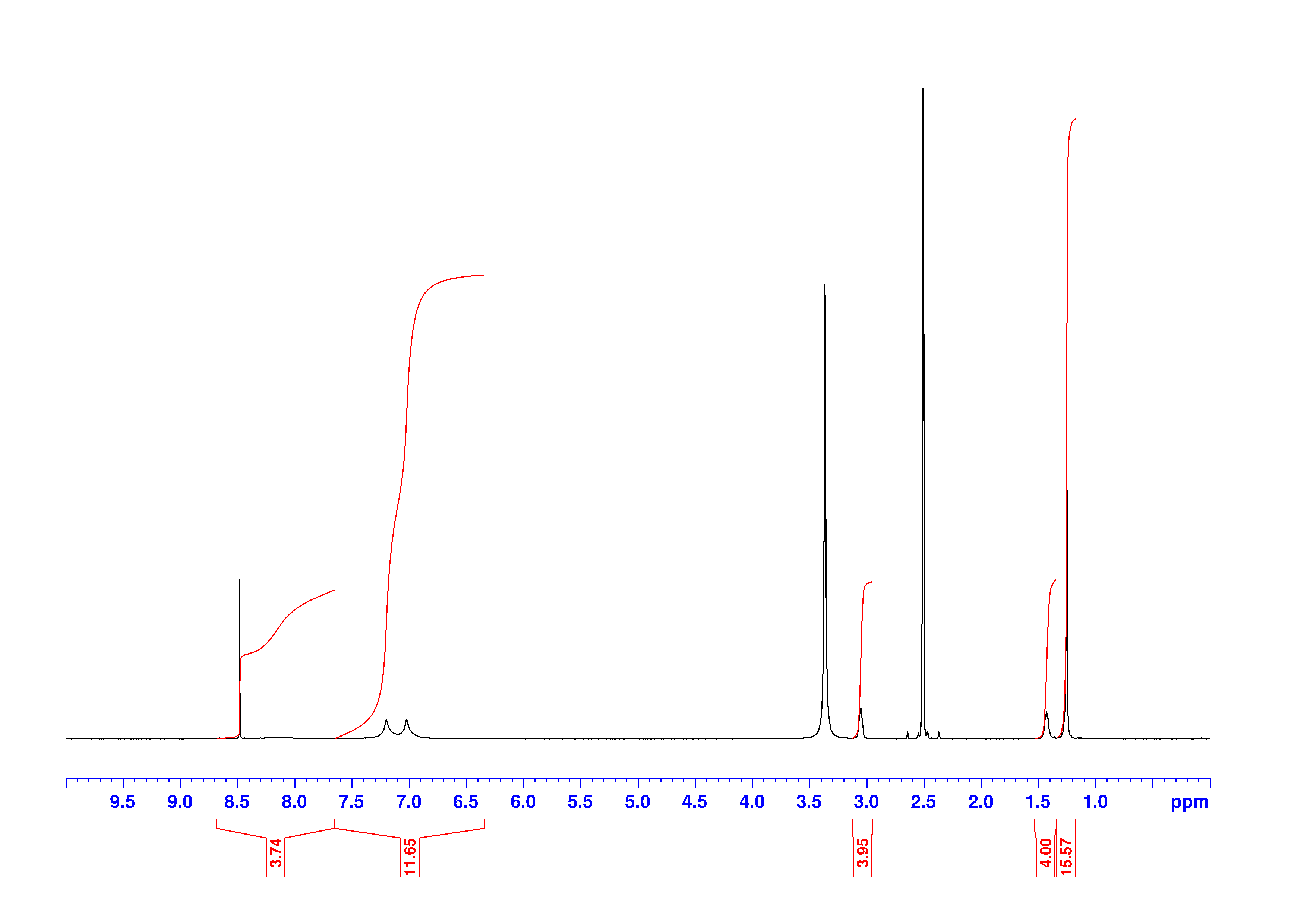


**^13^C NMR spectrum of isotopically labelled LCC-12 in DMSO-*d_6_***


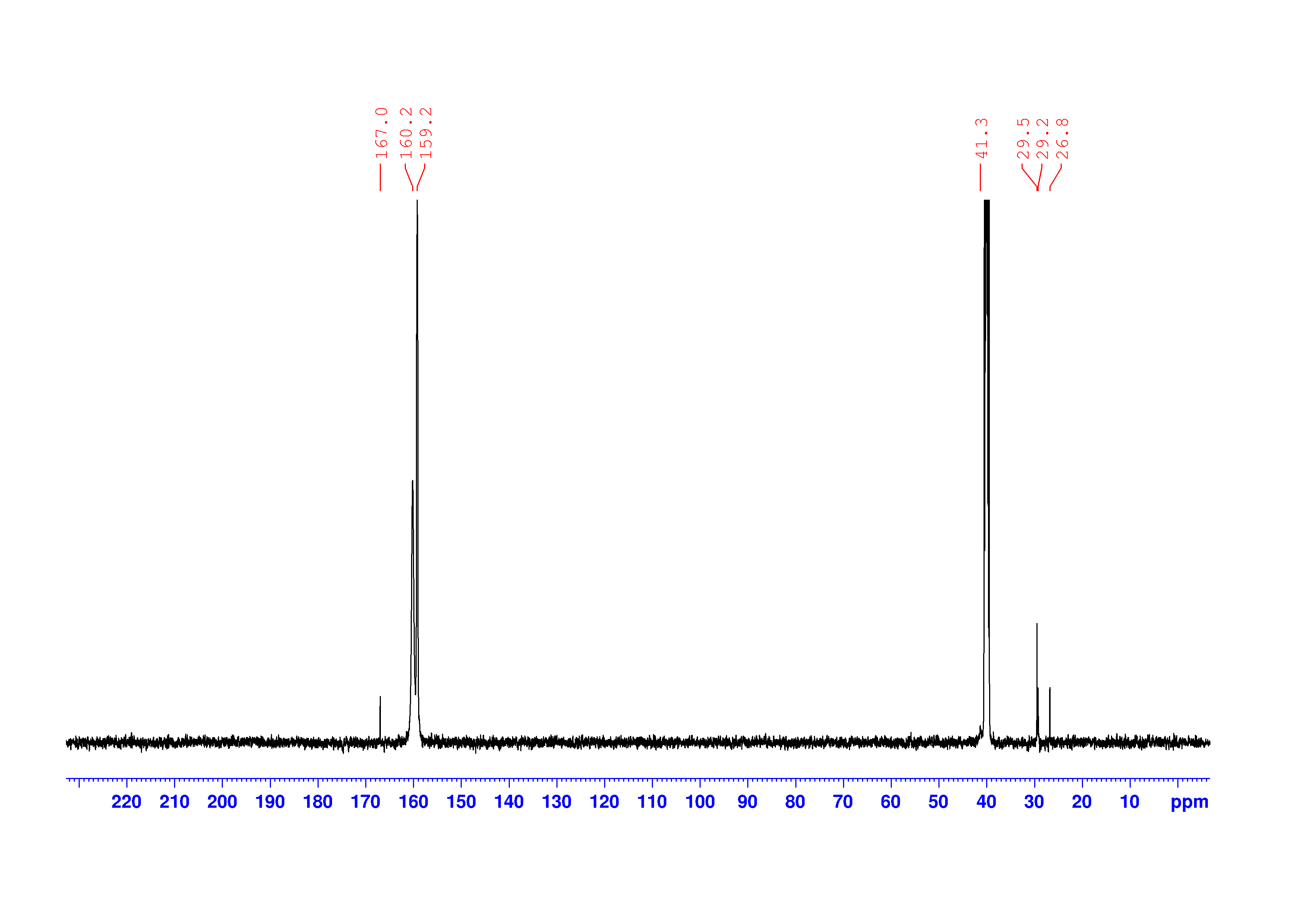


**^15^N NMR spectrum of isotopically labelled LCC-12 in DMSO-*d_6_***

**
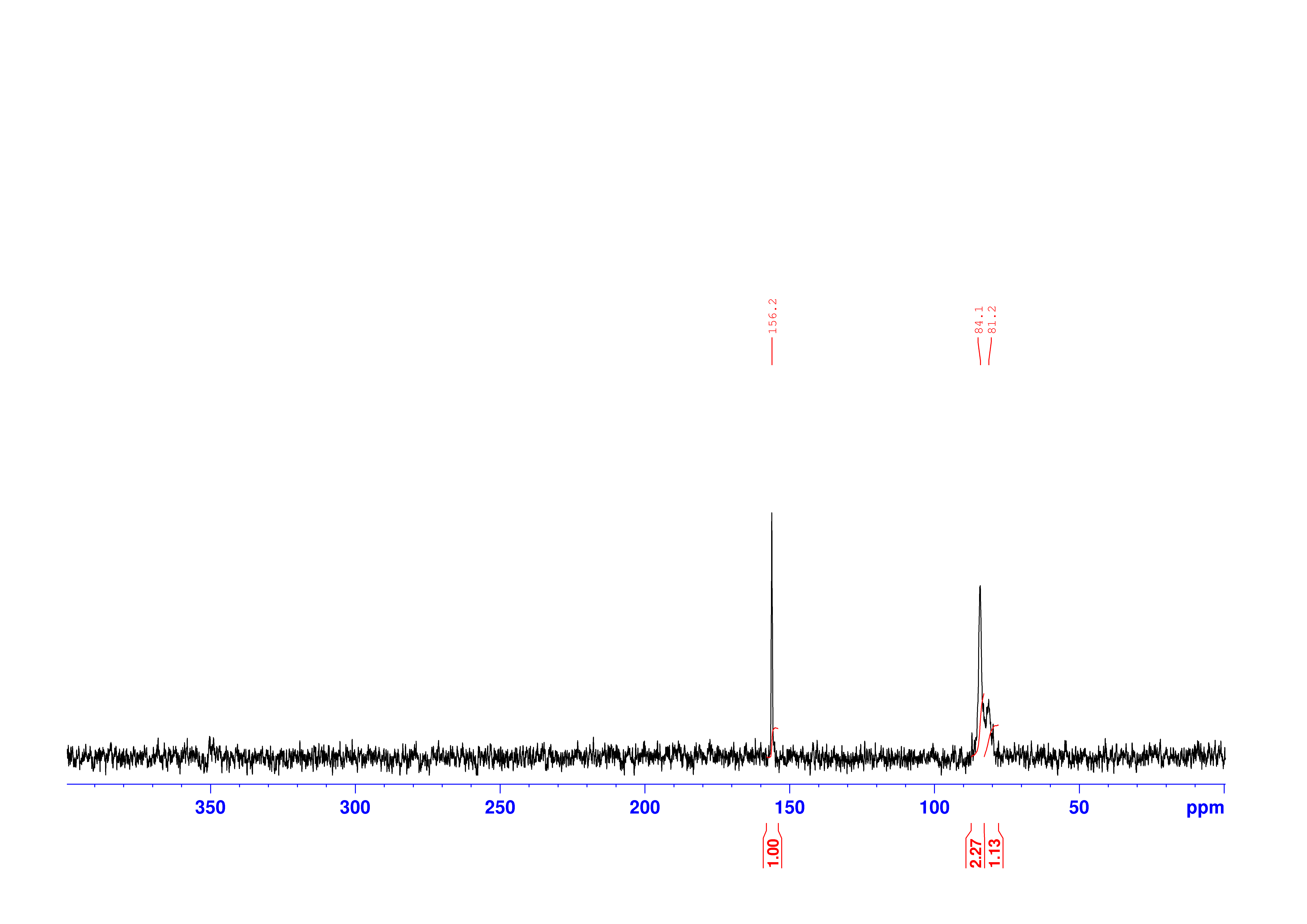
**

**^1^H NMR spectrum of HA, TBA-HA and meth-HA in D_2_O (0.125 M NaOD)**


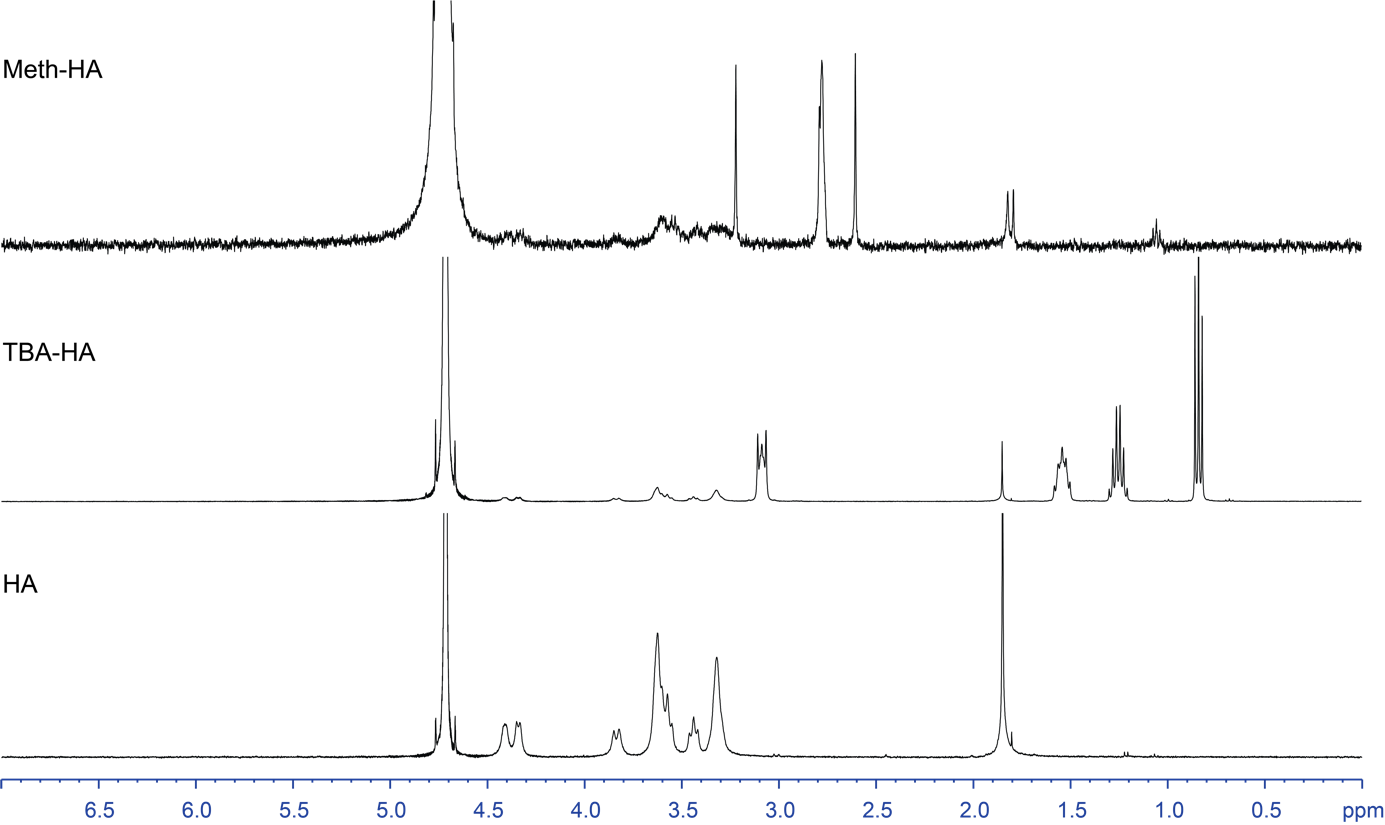


**^1^H NMR spectrum of trientine alkyne in methanol-*d*_4_**

**
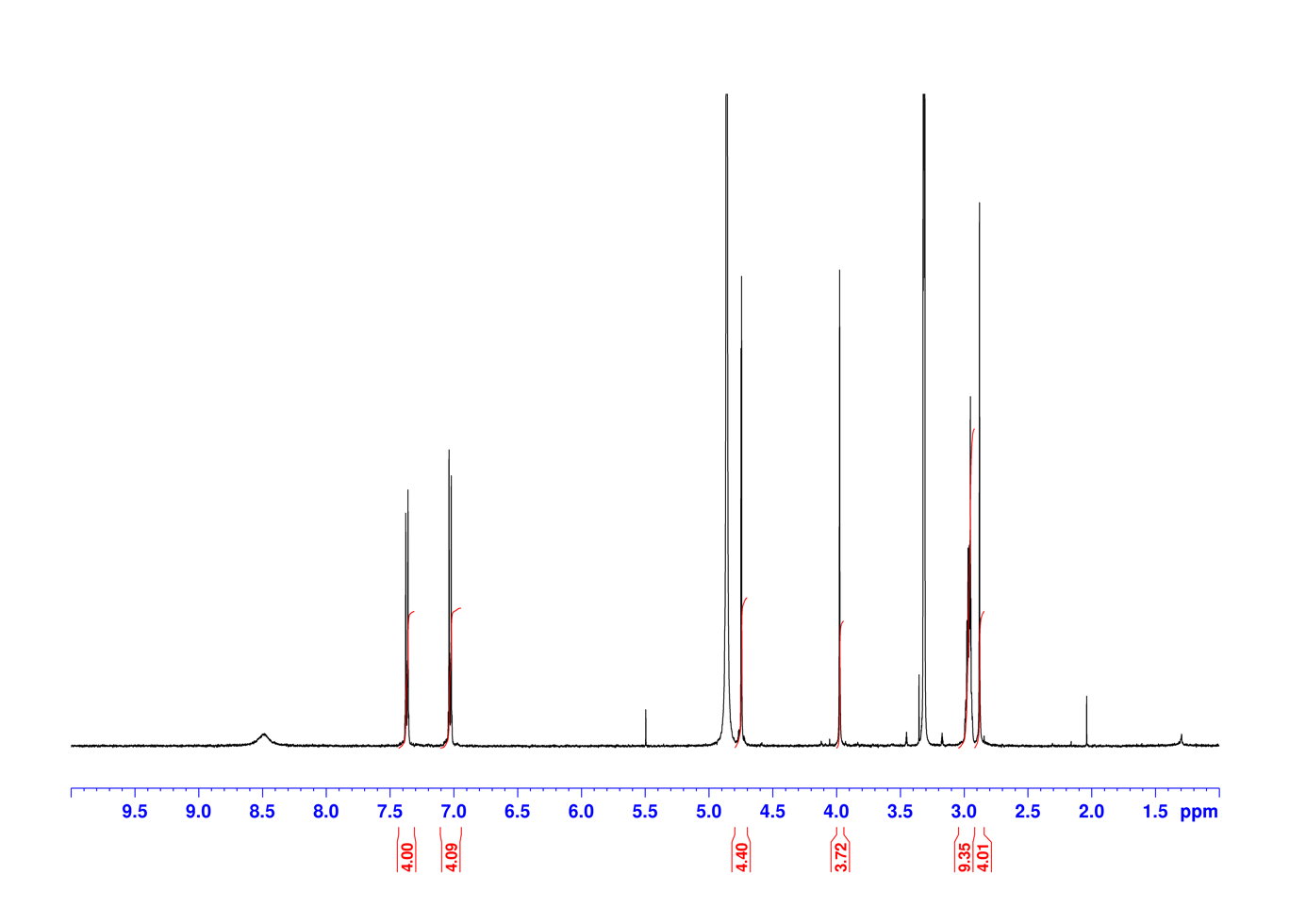
**

**^13^C NMR spectrum of trientine alkyne in methanol-*d*_4_**


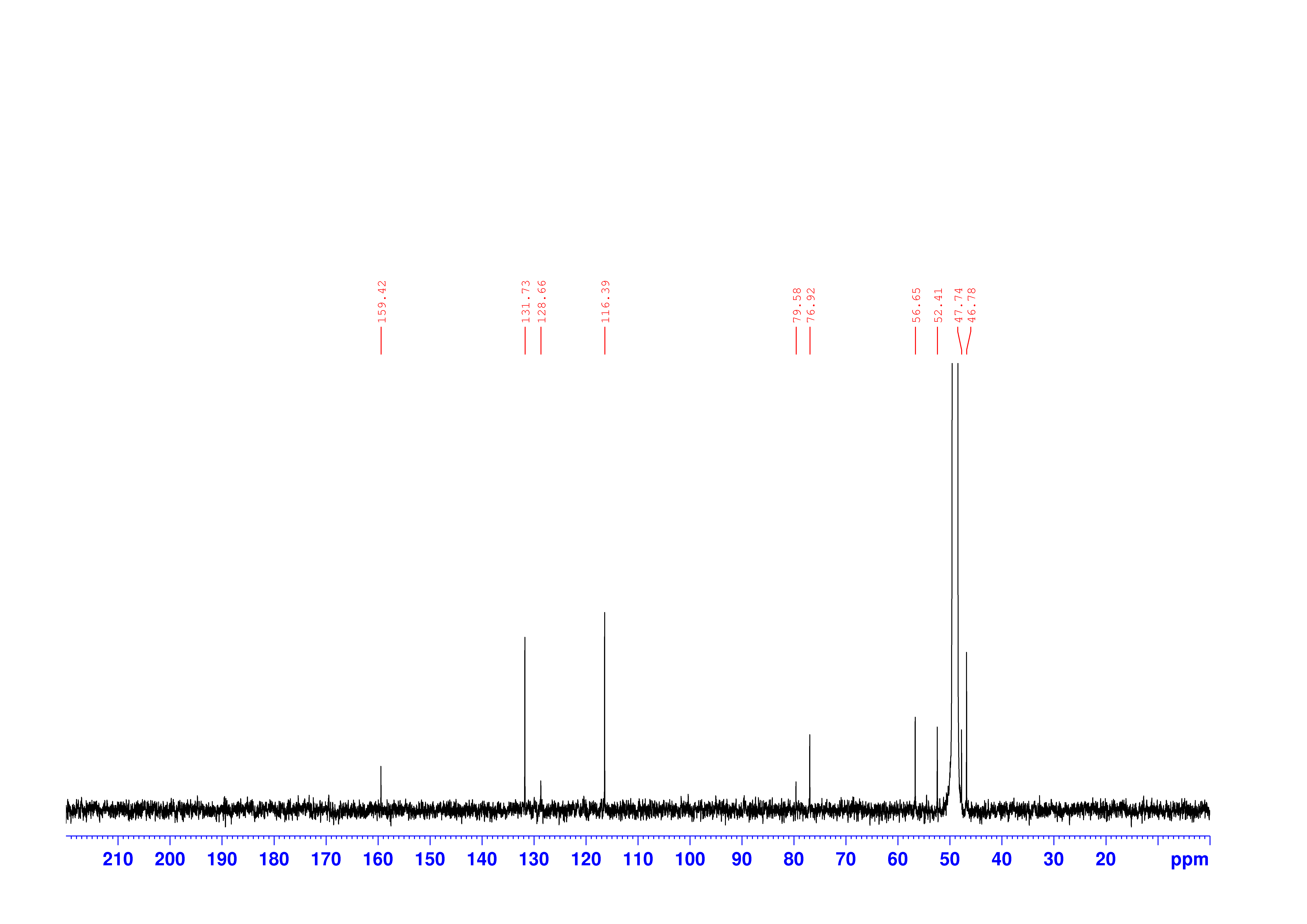

Supplement: Supplementary file 1 — This file contains Supplementary Table legends; sequences for RNA interference and genome-editing experiments; flow cytometry gating strategies; full western blots; and NMR spectra. [file 41586_2023_6017_MOESM1_ESM.docx]
