# Supplementary material for: A druggable copper-signalling pathway that drives inflammation
Source: Nature. 2023 Apr 26;617(7960):386–94. doi: 10.1038/s41586-023-06017-4 (PMC10131557; doi:10.1038/s41586-023-06017-4)
Supplement: Supplementary file 2 — Reporting Summary [file 41586_2023_6017_MOESM2_ESM.pdf]

nature portfolio

Corresponding author(s): Raphaël Rodriguez

Last updated by author(s): Mar 9, 2023

Reporting Summary

Nature Portfolio wishes to improve the reproducibility of the work that we publish. This form provides structure for consistency and transparency in reporting. For further information on Nature Portfolio policies, see our Editorial Policies and the Editorial Policy Checklist.

Statistics

For all statistical analyses, confirm that the following items are present in the figure legend, table legend, main text, or Methods section.

n/a	Confirmed	
☐	☒	The exact sample size (*n*) for each experimental group/condition, given as a discrete number and unit of measurement
☐	☒	A statement on whether measurements were taken from distinct samples or whether the same sample was measured repeatedly
☐	☒	The statistical test(s) used AND whether they are one- or two-sided<br>*Only common tests should be described solely by name; describe more complex techniques in the Methods section.*
☐	☒	A description of all covariates tested
☐	☒	A description of any assumptions or corrections, such as tests of normality and adjustment for multiple comparisons
☐	☒	A full description of the statistical parameters including central tendency (e.g. means) or other basic estimates (e.g. regression coefficient) AND variation (e.g. standard deviation) or associated estimates of uncertainty (e.g. confidence intervals)
☐	☒	For null hypothesis testing, the test statistic (e.g. *F*, *t*, *r*) with confidence intervals, effect sizes, degrees of freedom and *P* value noted<br>*Give P values as exact values whenever suitable.*
☒	☐	For Bayesian analysis, information on the choice of priors and Markov chain Monte Carlo settings
☒	☐	For hierarchical and complex designs, identification of the appropriate level for tests and full reporting of outcomes
☐	☒	Estimates of effect sizes (e.g. Cohen's *d*, Pearson's *r*), indicating how they were calculated

Our web collection on statistics for biologists contains articles on many of the points above.

Software and code

Policy information about availability of computer code

Data collection

No software was used

Data analysis

Code availability:
Analysis scripts for RNA-seq and ChIP-seq data are available at https://github.com/bioinfo-pf-curie/MDMmetals.

Softwares:
Flow cytometry
*FlowJo software v 10.8.2

Imaging
*Prism 8.2.0

Quantitative metabolomics
*Agilent Mass Hunter quantitative software B.07.01
*Agilent Mass Hunter quantitative software B.10.1

Quantitative proteomics
*Proteome Discoverer (version 2.4), Thermo Scientific
*myProMS (v3.9.3) (https://github.com/bioinfo-pf-curie/myproms), Poullet P et al., 2007

RNA-seq

*Raw sequencing reads were first checked for quality with Fastqc (0.11.8) and trimmed for adapter sequences with the trimGalore (0.6.2) software
*Trimmed reads were then aligned on the human hg38 reference genome using the STAR mapper (2.6.1b), up to the generation of a raw count table per gene (GENCODE annotation v29)
*The bioinformatics pipelines used for these tasks are available online (rawqc v2.1.0: https://github.com/bioinfo-pf-curie/raw-qc, RNA-seq v3.1.4: https://github.com/bioinfo-pf-curie/RNA-seq)
*Counts were normalized using TMM normalization from edgeR (v 3.30.3)
*Differential expression was assessed with the limma voom framework (v 3.44.3)
*Enrichment analysis from differentially expressed genes has been performed using the enrichGO function from clusterProfiler package v3.16.1.

ChIP-seq
ChIP-seq data processing and quality controls have been performed with the Institut Curie ChIP-seq Nextflow pipeline (1.0.6) available at https://github.com/bioinfo-pf-curie/ChIP-seq

Illustrations
*FIJI 2.0.0-rc-69/1.52n
*Prism 8.2.0
*Adobe Illustrator 26.0.2
*biorender.com

For manuscripts utilizing custom algorithms or software that are central to the research but not yet described in published literature, software must be made available to editors and reviewers. We strongly encourage code deposition in a community repository (e.g. GitHub). See the Nature Portfolio guidelines for submitting code & software for further information.

Data

Policy information about availability of data

All manuscripts must include a data availability statement. This statement should provide the following information, where applicable:

- Accession codes, unique identifiers, or web links for publicly available datasets
- A description of any restrictions on data availability
- For clinical datasets or third party data, please ensure that the statement adheres to our policy

Data availability:
RNA-seq and ChIP-seq data are available on the National Center for Biotechnology Information website with accession reference GSE160864 (go to: https://www.ncbi.nlm.nih.gov/geo/query/acc.cgi?acc=GSE160864). The mass spectrometry proteomics raw data have been deposited to the ProteomeXchange Consortium via the PRIDE partner repository with the dataset identifier PXD038612. The donor number corresponds to the order of blood collection.

Publicly available datasets:
* mouse mm10 reference genome
* human hg38 reference genome

Human research participants

Policy information about studies involving human research participants and Sex and Gender in Research.

Reporting on sex and gender	Peripheral blood samples were collected from 128 anonymous donors (Etablissement Français du Sang, EFS).
Population characteristics	Not available for anonymous donors.<br><br>According to the 2016 activity report of "Etablissement Français du Sang", half of the donors are under 40 years old, and consist of 52% women and 48% men.
Recruitment	It is not applicable. The blood samples are not selected but donated on a random basis according to availability.
Ethics oversight	The use of EFS blood samples from anonymous donors was approved by the Institut National de la Santé et de la Recherche Médicale committee. Written consent was obtained from all the donors.

Note that full information on the approval of the study protocol must also be provided in the manuscript.

Field-specific reporting

Please select the one below that is the best fit for your research. If you are not sure, read the appropriate sections before making your selection.

☒ Life sciences ☐ Behavioural & social sciences ☐ Ecological, evolutionary & environmental sciences

For a reference copy of the document with all sections, see nature.com/documents/nr-reporting-summary-flat.pdf

Life sciences study design

All studies must disclose on these points even when the disclosure is negative.

Sample size	No prior sample size calculation was performed.<br>A minimum of three independent experiments was done to be able to perform meaningful statistical analyses.
Data exclusions	No data were excluded from the analyses.
Replication	All attemps at replication were successful as stated in the reporting summary.
Randomization	This applies only to experiments involving mice with experimental groups. Consequently, randomization was made for these experiments.
Blinding	Data were blinded for ICP-MS data collection. For other data collection, blinding was not performed.

Reporting for specific materials, systems and methods

We require information from authors about some types of materials, experimental systems and methods used in many studies. Here, indicate whether each material, system or method listed is relevant to your study. If you are not sure if a list item applies to your research, read the appropriate section before selecting a response.

Materials & experimental systems

n/a	Involved in the study
☐	☒ Antibodies
☐	☒ Eukaryotic cell lines
☒	☐ Palaeontology and archaeology
☐	☒ Animals and other organisms
☒	☐ Clinical data
☒	☐ Dual use research of concern

Methods

n/a	Involved in the study
☐	☒ ChIP-seq
☐	☒ Flow cytometry
☒	☐ MRI-based neuroimaging

Antibodies

Antibodies used

WB: western blot, FC: flow cytometry, FM: fluorescence microscopy, NS: NanoSIMS, ChIP: ChIP-seq. Hu: used for human samples. Ms: used for mouse samples. Dilutions are indicated. Any antibody validation by manufacturers is indicated and can be found on the manufacturers' websites. Our antibody validation by knockdown (kd) and/or knockout (ko) strategies as described in the manuscript for relevant antibodies is indicated.

Primary: ALKBH1 (Abcam, ab195376, clone EPR19215, Lot GR262105-2, WB 1:1000, Hu, Ms, ko validated by manufacturer), AMP-activated protein kinase subunit alpha (AMPKalpha, Cell Signaling, 2532S, Lot 21, WB 1:1000, Hu), P-AMPKalpha phosphorylated on Thr172 (P-AMPKalpha, Cell Signaling, 2535S, Lot 27, WB 1:1000, Hu), ATF2 (Abcam, ab32160, clone E243, Lot GR3430555-1, WB 1:1000, Hu), ATF2 (Proteintech, 14834-1-AP, WB 1:1000, Ms, kd/ko validated by manufacturer), ATP7A (Santa Cruz Biotechnology, sc-376467, clone D-9, Lot J2821, WB 1:200, Hu), ATP7A (Novus Biologicals, NBP2-59376, clone S60-4, WB 1:1000, Ms), ATP7B (Santa Cruz Biotechnology, sc-373964, clone A-11, Lot I2719, WB 1:200, Hu, Ms), Catalase (Cell Signaling, 12980T, clone D4P7B, Lot 3, WB 1:1000, Hu), CCL2/Mcp1 (Proteintech, 66272-1-Ig, clone 1B9F7, WB 1:1000, Hu), CD11b-Pacific Blue (BioLegend, 101224, clone M1/70, Lot B350151, B323654 and B323653, FC 1:800, Ms), CD14-Krome Orange (Beckman Coulter, B01175, clone RMO/52, Lot 200040, FC 1:100, Hu), CD16-Pacific Blue (Beckman Coulter, B36292, clone 3G8, Lot 200029, FC 1:100, Hu), CD3 (BioLegend, 317326, clone OKT3, Lot B372352, T cell activation, 2.5 µg/mL, Hu), CD25-BV711 (BioLegend, 302636, clone BC96, Lot B281779, FC 1:100, Hu), CD28 (BioLegend, 302934, clone CD28.2, Lot 374639, T cell activation, 2.5 µg/mL, Hu), CD40-APC (BioLegend, 124612, clone 3/23, Lot B309981, FC 1:200, Ms), CD40-BV510 (BioLegend, 334329, clone 5C3, Lot 312131, FC 1:100, Hu), CD44 (Abcam, ab189524, Lot GR320797-13, GR3314218-16, clone EPR18668, WB 1:30000, Hu, Ms, ko/kd validated by us and manufacturer), CD44 (ThermoFisher Scientific, 701406, clone 19H8L4, Lot 1976318, FM 1:400, Hu), CD44-AF647 (Novus Biologicals, NB500-481AF647, clone MEM-263, Lot 118753, FC 1:100, Hu), CD44-AF647 (BioLegend, 103018, clone IM7, Lot B317762, FC 1:200, Ms), CD45-BV510 (BioLegend, 103138, clone 30-F11, Lot B362964, B322199, B333193, FC 1:200, Ms), CD64-FITC (BioLegend, 399505, clone S18012C, Lot 308498, FC 1:100, Hu), CD66b-Pe/Cy7 (BioLegend, 305115, clone G10F5, Lot 283925, FC 1:100, Hu), CD69-PerCP (BioLegend, 310928, clone FN50, Lot B290414, FC 1:100, Hu), CD80-AF700 (BD Biosciences, 561133, clone 307.4, Lot 1060235, FC 1:100, Hu), CD83-PE (BioLegend, 305307, clone HB15e, Lot B303073, FC 1:100, Hu), CD86-PE (BioLegend, 105007, clone GL-1, Lot B318893, FC 1:200, Ms), CD86-PE/Cy7 (BD Biosciences, 561128, clone 2331 (FUN-1), Lot 1309531, FC 1:33, Hu), CD109 (Santa Cruz Biotechnology, sc-271085, clone C-9, Lot E1018, WB 1:200, Hu), CD109 (Biotechne, AF7717-SP, WB 1:1000, Ms), CD163-PE (BD Biosciences, 556018, clone GHI/61, Lot 9143793, FC 1:100, Hu), CD170 (Siglec-F)-PEeFluor 610 (eBioscience, 61-1702-80, clone 1RNM44N, Lot 2472220 and 2152352, FC 1:200, Ms), cGAS (Cell Signaling, 15102, clone D1D3G, Lot 4, WB 1:1000, Hu), cGAS (Cell Signaling, 31659, clone D3O8O, Lot 3, WB 1:1000, Ms), CLOCK (Proteintech, 18094-1-AP, WB 1:1000, Hu, kd/ko validated by manufacturer), CLOCK (Abcam, ab3517, WB 1:1000, Ms), Copper transporter 1 (Ctr1, Abcam, ab129067, clone EPR7936, Lot GR3414582-4 and GR81444-2, WB 1:1000, Hu, kd validated by us), Copper transporter 2 (Ctr2, Novus Biologicals, NBP1-05199SS, WB 1:1000, Hu), Copper transporter 2 (Ctr2, Novus Biologicals, NBP1-85512, Lot R05901, FM 1:400, Hu), Copper transporter 2 (Biorybt, orb182668, Lot BR2373, WB 1:1000, Hu) COX IV (Abcam, ab16056, Lot GR320655-1, FM 1:400, Hu), CREM (Proteintech, 12131-1-AP, WB 1:1000, Hu),

Cytochrome c (Cyt c, Cell Signaling, 12963S, clone 6H2.B4, Lot 1 and 2, FM 1:400, NS 1:400, Hu), Divalent Metal Transporter 1 (DMT1, Abcam, ab55735, clone 4C6, Lot GR3243346-1, WB 1:1000, Hu, kd validated by us), Drosophila spike-in antibody (Active Motif, 61686, Lot 23521010, ChIP 50 ng per condition), E-cadherin (Cell Signaling, 3195, clone 24E10, Lot 15, WB 1:1000, Hu), E-cadherin (BD Biosciences, 610181, clone 36, Lot 7187865, WB 1:1000, Ms), F4/80-BV605 (BioLegend, 123133, clone BM8, Lot B362524, B309659, B331465 and B339746, FC 1:100, Ms), F4/80-PE (TONBO, TNB50-4801-U100, clone BM8.1, Lot C4801060619503, FC 1:100, Ms), Fibronectin (Sigma-Aldrich, F0791, clone IST-3, Lot 026M4781V, WB 1:1000, Hu, Ms), FTO (Proteintech, 27226-1-AP, WB 1:1000, Hu, Ms, kd/ko validated by manufacturer), H3 (Cell Signaling, 9715S, Lot 23, FM, WB 1:1000, Hu, Ms), H3K4me3 (Diagenode, C15410003-50, Lot A8034D, FM 1:400, Hu, dot blot validation by manufacturer), H3K9ac (Cell Signaling, 9649S, clone C5B11, Lot 13, FM, WB, ChIP 6µL per 1×106 cells, Hu, Ms, validated with SimpleChIP Enzymatic Chromatin IP by manufacturer), H3K9me2 (Cell Signaling, 4658S, clone D84B4, Lot 10, FM 1:400, WB 1:1000, ChIP 6µL per 1×106 cells, Hu, Ms, validated with SimpleChIP Enzymatic Chromatin IP by manufacturer), H3K9me3 (Cell Signaling, 13969S, clone D4W1U, Lot 3, FM 1:400, Hu, validated with SimpleChIP Enzymatic Chromatin IP by manufacturer), H3K14ac (Cell Signaling, 7627S, clone D4B90, Lot 6, FM, WB 1:1000, ChIP 6µL per 1×106 cells, Hu, Ms, validated with SimpleChIP Enzymatic Chromatin IP by manufacturer), H3K27ac (Cell Signaling, 8173S, clone D5E4, Lot 8, FM, WB 1:1000, ChIP 6µL per 1×106 cells, Hu, Ms, validated with SimpleChIP Enzymatic Chromatin IP by manufacturer), H3K27me3 (Cell Signaling, 9733S, clone C36B11, Lot 19, FM 1:400, WB 1:1000, ChIP 6µL per 1×106 cells, Hu, Ms, validated with SimpleChIP Enzymatic Chromatin IP by manufacturer), H3K36me2 (Abcam, ab9049, Lot GR3258133-1, FM 1:400, Hu), Hyaluronan synthase 1 (HAS1, Novus Biologicals, NBP1-51635, clone 3E10, Lot 141031, WB 1:1000, Hu), HAS1 (Sigma-Aldrich, SAB4300848, Lot 492637613, WB 1:1000, Ms), Hyaluronan synthase 2 (HAS2, Abcam, ab140671, clone 4E7, Lot GR3212928-2, WB 1:1000, Hu), HAS2 (Santa Cruz Biotechnology, sc-514737, clone A-7, WB 1:200, Ms), Hyaluronan synthase 3 (HAS3, Abcam, ab154104, Lot GR113715-12, WB 1:1000, Hu), HAS3 (Proteintech, 15609-1-AP, WB 1:1000, Ms, ko/kd validated by us and manufacturer), HAT1 (Proteintech, 11432-1-AP, WB 1:1000, Hu, Ms, ko/kd validated by us and manufacturer), 5-Hydroxymethylcytosine (5hmC, Active Motif, 39069, Lot 23720003, FM 1:400, Hu, dot blot validated by manufacturer), I-A/I-E-AF700 (BioLegend, 107622, clone M5/114.15.2, Lot B313251, FC 1:400, Ms), IRAK4 (Cell Signaling, 4363T, Lot 5, WB 1:1000, Hu, Ms), JAK2 (Cell Signaling, 3230T, clone D2E12, WB 1:1000, Lot 13, Hu, Ms), JMJD6 (Abcam, ab64575, Lot GR3441511-1, WB 1:1000, Hu, Ms), KAT2B/PCAF (Cell Signaling, 3378T, clone C14G9, Lot 2, WB 1:1000, Hu, Ms, validated with SimpleChIP Enzymatic Chromatin IP by manufacturer), KAT3A/CREBBP (Abcam, ab2832, Lot GR3360262-6, WB 1:1000, Hu, Ms), KAT5/Tip60 (Santa Cruz Biotechnology, sc-166323, clone C-7, Lot 166323, WB 1:200, Hu, Ms), KAT6A/MOZ (Santa Cruz Biotechnology, sc-293283, clone 4D8, Lot F0420, WB 1:200, Hu), KAT6A/MOZ (Invitrogen, PA5-103467, Lot XH3653004, WB 1:1000, Ms), KAT8/MOF (Proteintech, 13842-1-AP, kd/ko validated by manufacturer, WB 1:1000, Hu, Ms), KDM2A (Abcam, ab191387, clone EPR18602, Lot GR3330146-4, WB 1:1000, Hu, Ms, ko validated by manufacturer), KDM5A/Jarid1a (Cell Signaling, 3876S, clone D28B10, Lot 5, WB 1:1000, Hu, Ms), KDM5B/Jarid1b (Cell Signaling, 3273T, Lot 3, WB 1:1000, Hu), KDM5B/Jarid1b (Abcam, ab181089, clone EPR12794, WB 1:1000, Ms, ko validated by manufacturer), KDM5C/Jarid1c (Cell Signaling, 5361, clone D29B9, Lot 1, 5361, WB 1:1000, Hu, Ms), KDM6A/UTX (Cell Signaling, 33510S, clone D3Q1l, Lot 4, WB 1:1000, Hu, Ms), KDM6B (Abcam, ab169197, WB 1:1000, Hu), KDM6B/JMJD3 (Cell Signaling, 3457, WB 1:1000, Ms), KDM7A (Invitrogen, PA5-96987, Lot UI2838718, WB 1:1000, Hu, Ms), Ly6C-PerCP/Cy5.5 (BioLegend, 128012, clone HK1.4, Lot B363119 and B310463, FC 1:200, Ms), Ly6G-PE-Cy7 (BioLegend, 127618, clone 1A8, Lot B288785 and B351626, FC 1:200, Ms), Ly6G-AF647 (BioLegend, 127610, clone 1A8, Lot B2559839, FC 1:200, Ms), Lysosome-associated membrane protein 2 (LAMP2, Abcam, ab25631, clone H4B4, FM 1:400, Hu), NOS2-APC (eBioscience, 17-5920-82, clone CXNFT, Lot 2154045, FC 1:100, Ms), SLC25A3 (Santa Cruz Biotechnology, sc-376742, clone F-1, Lot H2313, WB 1:200, FM 1:100, Hu, kd validated by us), SLC25A3 (Abcam, ab89117, Lot 1015892-1, FM 1:400, Hu), SLC25A37 (MyBiosource, MBS9210193, clone ID: RB24153, Lot SA100524AR, WB 1:1000, FM 1:400, Hu, kd validated by us), Slug (Cell Signaling, 9585S, clone C19G7, Lot 6, WB 1:1000, Hu, Ms), STAT1 (Cell Signaling, 14994T, clone D1K9Y, Lot 8, WB 1:1000, Hu, Ms, validated with SimpleChIP Enzymatic Chromatin IP by manufacturer), STAT2 (Abcam, ab32367, clone Y141, Lot GR3294792-5, WB 1:1000, Hu, Ms, ko validated by manufacturer), Superoxide dismutase 2 (SOD2, Abcam, ab13534, Lot GR3345921-12 and GR33618-52, FM 1:400, WB 1:1000, Hu, Ms, kd validated by us), Tet methylcytosine dioxygenase 2 (TET2, Abcam, ab94580, Lot GR3243631-2, WB 1:1000, Hu, Ms), Tet methylcytosine dioxygenase 3 (TET3, Abcam, ab139311, Lot GR3314447-1, WB 1:1000, Hu, Ms), TRAF2 (Cell Signaling, 4724T, clone C192, Lot 2, WB 1:1000, Hu, Ms), Transferrin receptor 1 (TfR1, Invitrogen, 13-6800, clone H68.4, Lot VJ313549, WB 1:1000, Hu, kd validated by us), TfR1-APC-AF750 (Beckman Coulter, A89313, clone YDJ1.2.2, Lot 200060, FC 1:100, Hu), γ-Tubulin (Sigma-Aldrich, T5326, clone GTU-88, Source 0000128065, WB 1:1000, Hu, Ms, enhanced validation by manufacturer), Twist (Santa Cruz Biotechnology, sc-81417, clone Twist2C1a, Lot J2213, WB 1:200, Ms), Vimentin (Cell Signaling, 5741S, clone D21H3, Lot 8, WB 1:1000, Hu, Ms, ko validated by previous users according to manufacturer's website).

Secondary: Alexa-Fluor-488 anti-rabbit (Invitrogen, A-11070, Lot 2161039, FM 1:1000, Hu), Alexa-Fluor-594 anti-mouse (Invitrogen, A-11032, Lot 1826426, FM 1:1000, Hu), Alexa-Fluor-594 anti-rabbit (Invitrogen, A11072, Lot 1985650, FM 1:1000, Hu), Alexa-Fluor-647 anti-mouse (Invitrogen, A21237, Lot 1743738, FM 1:1000, Hu), Alexa-Fluor-647 anti-rabbit (Invitrogen, A21246, Lot 2418503, FM 1:1000, Hu), Donkey anti-Rabbit IgG-h+l HRP conjugated (Bethyl Laboratories, A120-108P, Lot 12 and 13), Goat anti-Mouse IgG h+l HRP conjugated (Bethyl Laboratories, A90-116P, Lot 41 and 44), 10 nM gold-nanoparticle-loaded anti-mouse (Abcam, ab27241, Lot GR274015-2, NS 1:200, Hu).

Validation

Any antibody validation by manufacturers is indicated and can be found on the respective websites and is specified in the antibody section. Our antibody validation by knockdown (kd) and/or knockout (ko) strategies, as described in the manuscript, is indicated.

Eukaryotic cell lines

Policy information about cell lines and Sex and Gender in Research

Cell line source(s)

Mouse pancreatic cancer cells FC1245 were a kind gift from Prof. D. Tuveson (Cold Spring Harbor)
Primary non-small cell lung circulating cancer cells (Celprogen, 36107-34CTC, Lot 219411, sex: female)

Authentication

FC1245 cells were a gift from the Tuveson laboratory. We did not perform additional characterization.

Primary non-small cell lung circulating cancer cells were obtained from Celprogen and are primary cells. We did not perform additional characterization.

Mycoplasma contamination	The cells were tested negative for mycoplasma contamination.
Commonly misidentified lines (See ICLAC register)	No commonly misidentified cell lines were used in this study.

Animals and other research organisms

Policy information about studies involving animals; ARRIVE guidelines recommended for reporting animal research, and Sex and Gender in Research

Laboratory animals	Survival assessment using the LPS mouse model was performed on 8-week-old male BALB/c mice. Other LPS experiments were performed on 8-week-old male BALB/c mice and 5-week-old male SWISS mice and experiments involving the CLP model were performed on 9-week-old male BALB/c mice. For experiments involving SARS-CoV-2, 8-week-old male K18-human ACE2 expressing C57BL/6 mice were used.<br><br>Mice were housed in state-of-the-art animal care facilities. Mice in all animal facilities were housed in ventilated cages (temperature 22 °C +/- 2 °C, humidity 55% +/- 10%) with free access to water and food on a 12 h light/dark cycle. Male littermates were randomly assigned to experimental groups throughout.
Wild animals	The study did not involve wild animals.
Reporting on sex	Experiments were performed on male mice.
Field-collected samples	The study did not involve samples collected from the field.
Ethics oversight	Survival assessment using the LPS mouse model was conducted at Fidelta Ltd (now Selvita) according to 2010/63/EU and National legislation regulating the use of laboratory animals in scientific research and for other purposes (Official Gazette 55/13). An institutional committee on animal research ethics (CARE-Zg) oversaw that animal-related procedures were not compromising the animal welfare.<br><br>Other experiments involving LPS and CLP mouse models were performed in accordance with French laws concerning animal experimentation (#2021072216346511) and approved by the Institutional Animal Care and Use Committee of Université de Saint-Quentin-en-Yvelines (C2EA-47). Mice were housed in a state-of-the-art animal care facility (2CARE, prefectural number agreement: A78-322-3, France).<br><br>A SARS-CoV-2 mouse model was used within the biosafety level 3 facility of the Institut Pasteur de Lille, after validation of the protocols by the local committee for the evaluation of the biological risks and complied with current national and institutional regulations and ethical guidelines (Institut Pasteur de Lille/B59-350009). The experimental protocols using animals were approved by the institutional ethical committee 'Comité d'Ethique en Experimentation Animale (CEEA) 75, Nord-Pas-de-Calais'. The animal study was authorized by the 'Education, Research and Innovation Ministry' under registration number APAFIS#25517-2020052608325772v3.<br><br>A SARS-CoV-2 mouse model was used within the biosafety level 3 facility of the University of Toulouse. This work was overseen by an Institutional Committee on Animal Research Ethics (License APAFIS#27729-2020101616517580 v3, Minister of Research, France (CEEA-001)), to ensure that animal-related procedures were not compromising the animal welfare.

Note that full information on the approval of the study protocol must also be provided in the manuscript.

ChIP-seq

Data deposition

☒ Confirm that both raw and final processed data have been deposited in a public database such as GEO.

☒ Confirm that you have deposited or provided access to graph files (e.g. BED files) for the called peaks.

Data access links *May remain private before publication.*	Data are available on the National Center for Biotechnology Information website with accession reference GSE160864 (https://www.ncbi.nlm.nih.gov/geo/query/acc.cgi?acc=GSE160864; enter token wvqxgwgojxcdboz into the box).
Files in database submission	Raw sequencing data as well as bigwig tracks have been shared on GEO.
Genome browser session (e.g. UCSC)	No UCSC session available.

Methodology

Replicates	All ChIP experiments have been performed using several donors.<br>For each donor, we have 3 conditions: naMDM, aMDM and aMDM LCC-12.
Sequencing depth	The sequencing depth varies from a type of histone mark to another. For active histone marks, an average of 50M read pairs have been sequenced. While around 150M read pairs have been sequenced for repressive histone marks.
Antibodies	Drosophila spike-in antibody (Active Motif, 61686, Lot 23521010, ChIP 50 ng per condition), H3K9ac (Cell Signaling, 9649S, clone

Antibodies

C5B11, Lot 13, FM, WB, ChIP 6µL per 1×106 cells, Hu, Ms, validated with SimpleChIP Enzymatic Chromatin IP by manufacturer), H3K9me2 (Cell Signaling, 4658S, clone D84B4, Lot 10, FM 1:400, WB 1:1000, ChIP 6µL per 1×106 cells, Hu, Ms, validated with SimpleChIP Enzymatic Chromatin IP by manufacturer), H3K14ac (Cell Signaling, 7627S, clone D4B90, Lot 6, FM, WB 1:1000, ChIP 6µL per 1×106 cells, Hu, Ms, validated with SimpleChIP Enzymatic Chromatin IP by manufacturer), H3K27ac (Cell Signaling, 8173S, clone D5E4, Lot 8, FM, WB 1:1000, ChIP 6µL per 1×106 cells, Hu, Ms, validated with SimpleChIP Enzymatic Chromatin IP by manufacturer), H3K27me3 (Cell Signaling, 9733S, clone C36B11, Lot 19, FM 1:400, WB 1:1000, ChIP 6µL per 1×106 cells, Hu, Ms, validated with SimpleChIP Enzymatic Chromatin IP by manufacturer).

Peak calling parameters

No peak calling was in this study. As the goal was mainly to integrate histone modification and gene expression, the ChIP-seq density signal has been extracted around gene TSS (+-2kb) for active histone marks and along the gene body for repressive marks.

Data quality

All samples passed quality checks, with more than 80% of aligned reads on the genome and a significant enrichment (fingerprint).

Software

The bioinformatics pipeline used to process the ChIP-seq data is available online at https://github.com/bioinfo-pf-curie/ChIP-seq.

Flow Cytometry

Plots

Confirm that:

☒ The axis labels state the marker and fluorochrome used (e.g. CD4-FITC).

☒ The axis scales are clearly visible. Include numbers along axes only for bottom left plot of group (a 'group' is an analysis of identical markers).

☒ All plots are contour plots with outliers or pseudocolor plots.

☒ A numerical value for number of cells or percentage (with statistics) is provided.

Methodology

Sample preparation

Flow cytometry on human immune cells: cells were washed with ice-cold 1× PBS, incubated with Fc block (Human TruStain FcX, BioLegend, 422302, 1:20) for 15 min, subsequently incubated with antibodies for 20 min at 4 °C in 1× PBS/ 0.5% bovine serum albumin (BSA) and then washed before analysis using a flow cytometer (BD LSRFortessa X-20). Cells were analyzed with the corresponding antibody panels.

Flow cytometry on primary non-small cell lung circulating cancer cells and FC1245 cells: cells were harvested by trypsinization using Trypsin/EDTA (GIBCO, TRYPGIB01), washed with 1× PBS and antibody incubation was performed for 20 mins at 4 °C in 1× PBS/10% FBS. Cells were washed and analyzed using a flow cytometer (BD Accurie C6).

Flow cytometry on LPS-induced severe inflammation mouse model: after euthanasia at 22 h post LPS challenge, the organs were perfused with PBS/EDTA (1 mL/g, 2mM, pH 7.4) and 10 mL of 1× PBS were injected in the peritoneum. The peritoneal liquid was then collected and centrifugated for 5 min at 1500 rpm. The pellet was resuspended in RPMI medium containing 2% fetal calf serum (Dutscher, S181H-100). The peritoneal liquid was then washed in 96-well plates at 2000 rpm for 2 min, and pellets were suspended in complete RPMI. For intracellular protein staining, samples were incubated for 2 h in brefeldin A (BFA, 5 ng/mL, Invitrogen, 00-4506-51) before surface staining with Fixable Viability Dye eFluor 780 (Invitrogen, 65-0865-14) followed by fluorochrome-conjugated antibodies (35 min at 4 °C). Samples incubated with BFA were fixed (Foxp3/ Transcription factor Fix/Perm 4X, Cell Signaling, 44931S) for 20 min at 4 °C and permeabilized (Flow Cytometry Perm Buffer 10X, TONBO, TNB-1213-L150) before intracellular staining. The antibodies used were as follows: CD11b-Pacific Blue (BioLegend, 101224), CD40-APC (BioLegend, 124612), CD45-BV510 (BioLegend, 103138), CD86-PE (BioLegend, 105007), CD170 (Siglec-F)-PEeFluor 610 (eBioscience, 61-1702-80), F4/80-BV605 (BioLegend, 123133), I-A/I-E-AF700 (BioLegend, 107622), Ly6C-PerCP/Cy5.5 (BioLegend, 128012) and Ly6G-PE/CY7 (BioLegend, 127618). For intracellular staining, NOS2-APC (eBioscience, 17-5920-82) was used. Small peritoneal macrophages (SPM) correspond to CD45+/IA-IE+/CD11b+/F4/80int/ SiglecF- cells. After washing with PBS or with Perm Buffer, data were acquired using an LSR Fortessa flow cytometer (BD Biosciences).

Flow cytometry on the CLP murine model: the sorting of SPM was done using the following antibodies: CD11b-Pacific Blue (BioLegend, 101224), F4/80-PE (TONBO, TNB50-4801-U100), Ly6C-PerCP/Cy5.5 (BioLegend, 128012) and Ly6G-AF647 (BioLegend, 127610). The sorted SPM corresponded to CD11b+/F4/80int/Ly6C-/Ly6G- cells.

Flow cytometry on SARS-CoV-2 mouse model: after a terminal anesthesia (ketamine 100 mg/kg and xylazine 10 mg/kg IP), lung tissues were harvested and homogenized in gentleMACS C Tubes (Miltenyi Biotec) containing 2.5 mL of RPMI 1640 medium (Gibco) and collagenase (2.5 mg/mL, Roche) using a gentleMACS Dissociator (Miltenyi Biotec). Lung tissues were further dissociated for 30 min at 37 °C under shaking, passed through a 70 µm cell strainer, and proceeded for red blood cell lysis before staining for cell sorting. Cells were stained with Fixable Viability Dye eFluor 780 (Invitrogen, 65-0865-14) followed by fluorochrome-conjugated antibodies (35 min at 4 °C). The antibodies used were as follows: CD11b-Pacific Blue (BioLegend, 101224), CD45-BV510 (BioLegend, 103138), CD170 (Siglec-F)-PEeFluor 610 (eBioscience, 61-1702-80), F4/80-BV605 (BioLegend, 123133). Alveolar macrophages (AM) correspond to CD45+/CD11bint/F4/80+/SiglecF+ cells and were isolated on a BD FACSAria Fusion.

Instrument

flow cytometers: BD LSRFortessa X-20, BD FACSAria and BD Accuri C6

Software

FlowJo software v 10.8.2

Cell population abundance

For human cells, cell sorting allowed to obtain a satisfactory purity.

Gating strategy

Murine small peritoneal macrophages (SPM) correspond to CD45+/IA-IE+/CD11b+/F4/80int/SiglecF- cells.
Alveolar macrophages (AM) correspond to CD45+/CD11bint/F4/80+/SiglecF+ cells. The gating strategies are detailed in the Supplementary Information.

☒ Tick this box to confirm that a figure exemplifying the gating strategy is provided in the Supplementary Information.
